# Hermite–Hadamard type inequalities for *F*-convex function involving fractional integrals

**DOI:** 10.1186/s13660-018-1950-1

**Published:** 2018-12-29

**Authors:** Pshtiwan Othman Mohammed, Mehmet Zeki Sarikaya

**Affiliations:** 1 Department of Mathematics, College of Education, University of Sulaimani, Sulaimani, Iraq; 2 Department of Mathematics, Faculty of Science and Arts, Düzce University, Düzce, Turkey

**Keywords:** 26A51, 26D15, 35A23, *F*-convex, $\lambda _{\varphi }$-preinvex, Integral inequalities

## Abstract

In this study, the family *F* and *F*-convex function are given with its properties. In view of this, we establish some new inequalities of Hermite–Hadamard type for differentiable function. Moreover, we establish some trapezoid type inequalities for functions whose second derivatives in absolute values are *F*-convex. We also show that through the notion of *F*-convex we can find some new Hermite–Hadamard type and trapezoid type inequalities for the Riemann–Liouville fractional integrals and classical integrals.

## Introduction

A function $f: I\subseteq \mathbb{R}\to \mathbb{R}$ is said to be convex on the interval *I*, if for all $x,y\in I$ and $t\in (0,1)$ it satisfies the following inequality:

1 $$\begin{aligned} f\bigl(tx+(1-t)y\bigr)\leq tf(x)+(1-t)f(y). \end{aligned}$$

Convex functions play an important role in the field of integral inequalities. For convex functions, many equalities and inequalities have been established, but one of the most important ones is the Hermite–Hadamard’ integral inequality, which is defined as follows [1]:

Let $f: I\subseteq \mathbb{R}\to \mathbb{R}$ be a convex function with $a< b$ and $a, b\in I$. Then the Hermite–Hadamard inequality is given by

2 $$\begin{aligned} f \biggl(\frac{a+b}{2} \biggr) &\leq \frac{1}{b-a} \int _{a}^{b} f(x)\,dx \leq \frac{f(a)+f(b)}{2}. \end{aligned}$$

In recent years, a number of mathematicians have devoted their efforts to generalizing, refining, counterparting, and extending the Hermite–Hadamard inequality (2) for different classes of convex functions and mappings. The Hermite–Hadamard inequality (2) is established for the classical integral, fractional integrals, conformable fractional integrals and most recently for generalized fractional integrals; see for details and applications [2–8] and the references therein.

The concepts of classical convex functions have been extended and generalized in several directions, such as quasi-convex [9], pseudo-convex [10], *MT*-convex [11] strongly convex [12], *ϵ*-convex [13], *s*-convex [14], *h*-convex [15], and $\lambda _{\varphi }$-preinvex [16]. Recently, Samet [17] has defined a new concept of convexity that depends on a certain function satisfying some axioms, generalizing different types of convexity, including *ϵ*-convex functions, *α*-convex functions, *h*-convex functions, and so on, as stated in the next section.

## Review of the family of $\mathcal{F}$

We address the family of $\mathcal{F}$ of mappings $F:\mathbb{R} \times \mathbb{R}\times \mathbb{R}\times [0,1]\to \mathbb{R}$ satisfying the following axioms:

1. If $u_{i}\in L^{1}(0,1)$, $i=1,2,3$, then, for every $\lambda \in [0,1]$, we have
  $$\begin{aligned} \int _{0}^{1} F\bigl(u_{1}(t),u_{2}(t),u_{3}(t), \lambda \bigr)\,dt=F \biggl( \int _{0}^{1} u_{1}(t)\,dt, \int _{0}^{1} u_{2}(t)\,dt, \int _{0}^{1} u_{3}(t)\,dt, \lambda \biggr). \end{aligned}$$
2. For every $u\in L^{1}(0,1)$, $w\in L^{\infty }(0,1)$ and $(z_{1},z_{2})\in \mathbb{R}^{2}$, we have
  $$\begin{aligned} \int _{0}^{1} F\bigl(w(t) u(t),w(t)z_{1},w(t)z_{2},t \bigr)\,dt=T_{F,w} \biggl( \int _{0}^{1} w(t) u(t)\,dt,z_{1},z_{2} \biggr), \end{aligned}$$
  where $T_{F,w}:\mathbb{R}\times \mathbb{R}\times \mathbb{R}\to \mathbb{R}$ is a function that depends on $(F,w)$, and it is nondecreasing with respect to the first variable.
3. For any $(w,u_{1},u_{2},u_{3})\in \mathbb{R}^{4}$, $u_{4} \in [0,1]$, we have
  $$\begin{aligned} wF(u_{1},u_{2},u_{3},u_{4})=F(wu_{1},wu_{2},wu_{3},u_{4})+L_{w}, \end{aligned}$$
  where $L_{w}\in \mathbb{R}$ is a constant that depends only on *w*.

### Definition 2.1

Let $f: [a,b]\to \mathbb{R}$, $(a,b)\in \mathbb{R}^{2}$, $a< b$, be a given function. We say that *f* is a convex function with respect to some $F\in \mathcal{F}$ (or *F*-convex function) iff

$$\begin{aligned} F\bigl(f\bigl(tx+(1-t)y\bigr),f(x),f(y),t\bigr)\leq 0, \quad (x,y,t)\in [a,b] \times [a,b] \times [0,1]. \end{aligned}$$

### Remark 1

Suppose that $(a,b)\in \mathbb{R}^{2}$ with $a< b$.

(i) Let $f:[a,b]\to \mathbb{R}$ be an *ε*-convex function, that is [18],
  $$\begin{aligned} f\bigl(tx+(1-t)y\bigr)\leq tf(x)+(1-t)f(y),\quad (x,y,t)\in [a,b]\times [a,b] \times [0,1]. \end{aligned}$$
  Define the functions $F:\mathbb{R}\times \mathbb{R}\times \mathbb{R} \times [0,1]\to \mathbb{R}$ by
  3 $$\begin{aligned} F(u_{1},u_{2},u_{3},u_{4})=u_{1}-u_{4}u_{2}-(1-u_{4})u_{3}- \varepsilon \end{aligned}$$
  and $T_{F,w}:\mathbb{R}\times \mathbb{R}\times \mathbb{R}\times [0,1] \to \mathbb{R}$ by
  4 $$\begin{aligned} T_{F,w}(u_{1},u_{2},u_{3})=u_{1}- \biggl( \int _{0}^{1} t w(t)\,dt \biggr)u _{2} - \biggl( \int _{0}^{1} (1-t)w(t)\,dt \biggr)u_{3}- \varepsilon . \end{aligned}$$
  For
  5 $$\begin{aligned} L_{w}=(1-w)\varepsilon , \end{aligned}$$
  it will be seen that $F\in \mathcal{F}$ and
  $$\begin{aligned} F\bigl(f\bigl(tx+(1-t)y\bigr),f(x),f(y),t\bigr) &=f\bigl(tx+(1-t)y \bigr)-tf(x)-(1-t)f(y)-\varepsilon \leq 0, \end{aligned}$$
  that is, *f* is an *F*-convex function. Particularly, taking $\varepsilon =0$ we show that if *f* is a convex function then *f* is an *F*-convex function with respect to *F* defined above.
(ii) Let $f:[a,b]\to \mathbb{R}$ be $\lambda _{\varphi }$-preinvex function according to *φ* and bifunction *η*, $0\leq \varphi \leq \frac{\pi }{2}$, $\lambda \in (0,\frac{1}{2} ]$, that is [16],
  $$\begin{aligned} &f \bigl(u+te^{i\varphi }\eta (v,u) \bigr) \\ &\quad \leq \frac{\sqrt{t}}{2 \sqrt{1-t}}f(v) + \frac{(1-\lambda )\sqrt{1-t}}{2\lambda \sqrt{t}}f(u), \quad (u,v,t) \in [a,b]\times [a,b]\times (0,1). \end{aligned}$$
  Define the functions $F:\mathbb{R}\times \mathbb{R}\times \mathbb{R} \times [0,1]\to \mathbb{R}$ by
  6 $$\begin{aligned} F(u_{1},u_{2},u_{3},u_{4})=u_{1}- \frac{\sqrt{u_{4}}}{2\sqrt{1-u _{4}}}u_{3} -\frac{(1-\lambda )\sqrt{1-u_{4}}}{2\lambda \sqrt{u _{4}}}u_{2} \end{aligned}$$
  and $T_{F,w}:\mathbb{R}\times \mathbb{R}\times \mathbb{R}\times [0,1] \to \mathbb{R}$ by
  7 $$\begin{aligned} \begin{aligned}[b] T_{F,w}(u_{1},u_{2},u_{3})&=u_{1}- \biggl( \int _{0}^{1} \frac{\sqrt{t}}{2 \sqrt{1-t}}w(t)\,dt \biggr)u_{3} \\ &\quad {}-\frac{1-\lambda }{\lambda } \biggl( \int _{0}^{1} \frac{\sqrt{1-t}}{2\sqrt{t}}w(t)\,dt \biggr)u_{2}. \end{aligned} \end{aligned}$$
  For $L_{w}=0$, it will be seen that $F\in \mathcal{F}$ and
  $$\begin{aligned} &F \bigl(f \bigl(u+te^{i\varphi }\eta (v,u) \bigr),f(u),f(v),t \bigr) \\ &\quad =f \bigl(u+te^{i\varphi }\eta (v,u) \bigr)-\frac{\sqrt{t}}{2 \sqrt{1-t}}f(v) - \frac{(1-\lambda )\sqrt{1-t}}{2\lambda \sqrt{t}}f(u)- \varepsilon \leq 0, \end{aligned}$$
  that is *f* is an *F*-convex function.
(iii) Let $h:I\to \mathbb{R}$ be a given function which is not identical to 0, where *I* is an interval in $\mathbb{R}$ such that $(0,1)\subseteq I$. Let $f:[a,b]\to [0,\infty )$ be an *h*-convex function, that is,
  $$\begin{aligned} f\bigl(tx+(1-t)y\bigr)\leq h(t)f(x)+\frac{1-\lambda }{\lambda }h(1-t)f(y), \quad (x,y,t) \in [a,b]\times [a,b]\times [0,1]. \end{aligned}$$
  Define the functions $F:\mathbb{R}\times \mathbb{R}\times \mathbb{R} \times [0,1]\to \mathbb{R}$ by
  8 $$\begin{aligned} F(u_{1},u_{2},u_{3},u_{4})=u_{1}-h(u_{4})u_{3}- \frac{1-\lambda }{ \lambda }h(1-u_{4})u_{2} \end{aligned}$$
  and $T_{F,w}:\mathbb{R}\times \mathbb{R}\times \mathbb{R}\times [0,1] \to \mathbb{R}$ by
  9 $$\begin{aligned} T_{F,w}(u_{1},u_{2},u_{3})=u_{1}- \biggl( \int _{0}^{1} h(t)w(t)\,dt \biggr)u _{3} - \frac{1-\lambda }{\lambda } \biggl( \int _{0}^{1} h(1-t)w(t)\,dt \biggr)u _{2}. \end{aligned}$$
  For $L_{w}=(1-w)\varepsilon $, it will be seen that $F\in \mathcal{F}$ and
  $$\begin{aligned} &F\bigl(f\bigl(tx+(1-t)y\bigr),f(x),f(y),t\bigr)\\ &\quad =f\bigl(tx+(1-t)y \bigr)-h(t)f(x)-\frac{1-\lambda }{ \lambda }h(1-t)f(y) -\varepsilon \leq 0, \end{aligned}$$
  that is *f* is an *F*-convex function.

Recently Samet [17] established some integral inequalities of Hermite–Hadamard type via *F*-convex functions.

### Theorem 1

([17, Theorem 3.1])

*Let* $f: [a,b]\to \mathbb{R}$, $(a,b)\in \mathbb{R}^{2}$, $a< b$, *be an* *F*-*convex function*, *for some* $F\in \mathcal{F}$. *Suppose that* $F\in L^{1}(a,b)$. *Then*

$$\begin{aligned} &F \biggl(f \biggl(\frac{a+b}{2} \biggr),\frac{1}{b-a} \int _{a}^{b} f(x)\,dx, \frac{1}{2} \biggr)\leq 0, \\ &T_{F,1} \biggl(\frac{1}{b-a} \int _{a}^{b} f(x)\,dx,f(a),f(b) \biggr) \leq 0. \end{aligned}$$

### Theorem 2

([17, Theorem 3.4])

*Let* $f: I^{o}\subseteq \mathbb{R}\to \mathbb{R}$ *be a differentiable mapping on* $I^{o}$, $(a,b)\in I^{o}\times I^{o}$, $a< b$. *Suppose that*

(i) $|f'|$ *is* *F*-*convex on* $[a,b]$, *for some* $F\in \mathcal{F;}$
(ii) *the function* $t\in (0,1)\to L_{w(t)}$ *belongs to* $L^{1}(a,b)$, *where* $w(t)=|1-2t|$.

*Then*

$$\begin{aligned} &T_{F,w} \biggl(\frac{2}{b-a} \biggl\vert \frac{f(a)+f(b)}{2}- \frac{1}{b-a} \int _{a}^{b} f(x)\,dx \biggr\vert , \bigl\vert f'(a) \bigr\vert , \bigl\vert f'(b) \bigr\vert \biggr)+ \int _{0}^{1} L _{w(t)}\,dt\leq 0. \end{aligned}$$

### Theorem 3

([17, Theorem 3.5])

*Let* $f: I^{o}\subseteq \mathbb{R}\to \mathbb{R}$ *be a differentiable mapping on* $I^{o}$, $(a,b)\in I^{o}\times I^{o}$, $a< b$ *and let* $p>1$. *Suppose that* $|f'|^{p/(p-1)}$ *is* *F*-*convex on* $[a,b]$, *for some* $F\in \mathcal{F}$ *and* $F\in L^{p/(p-1)}(a,b)$. *Then*

$$\begin{aligned} &T_{F,1} \bigl(A(p,f), \bigl\vert f'(a) \bigr\vert ^{\frac{p}{p-1}}, \bigl\vert f'(b) \bigr\vert ^{\frac{p}{p-1}} \bigr) \leq 0, \end{aligned}$$

*where*

$$\begin{aligned} A(p,f) &= \biggl(\frac{2}{b-a} \biggr)^{\frac{p}{p-1}}(p+1)^{ \frac{1}{p-1}} \biggl\vert \frac{f(a)+f(b)}{2}-\frac{1}{b-a} \int _{a}^{b} f(x)\,dx \biggr\vert ^{\frac{p}{p-1}}. \end{aligned}$$

As consequences of the above theorems, the author obtained some integral inequalities for *ε*-convexity, *α*-convexity, and *h*-convexity.

### Theorem 4

([17, Corollary 4.3])

*Let* $f: I^{o}\subseteq \mathbb{R}\to \mathbb{R}$ *be a differentiable mapping on* $I^{o}$, $(a,b)\in I^{o}\times I^{o}$, $a< b$. *Suppose that the function* $|f'|$ *is* *ε*-*convex on* $[a,b]$, $\varepsilon \geq 0$. *Then*

$$\begin{aligned} & \biggl\vert \frac{f(a)+f(b)}{2}-\frac{1}{b-a} \int _{a}^{b} f(x)\,dx \biggr\vert \leq (b-a) \biggl[\frac{ \vert f'(a) \vert + \vert f'(b) \vert }{8}+\frac{\varepsilon }{4} \biggr]. \end{aligned}$$

### Theorem 5

([17, Corollary 4.9])

*Let* $f: I^{o}\subseteq \mathbb{R}\to \mathbb{R}$ *be a differentiable mapping on* $I^{o}$, $(a,b)\in I^{o}\times I^{o}$, $a< b$. *Suppose that the function* $|f'|$ *is* *α*-*convex on* $[a,b]$, $\alpha \in (0,1]$. *Then*

$$\begin{aligned} & \biggl\vert \frac{f(a)+f(b)}{2}-\frac{1}{b-a} \int _{a}^{b} f(x)\,dx \biggr\vert \\ &\quad \leq \frac{(b-a)}{2(\alpha +1)(\alpha +2)} \biggl[ \bigl(2^{-\alpha }+ \alpha \bigr) \bigl\vert f'(a) \bigr\vert \frac{\alpha (\alpha +1)+2 (1-2^{-\alpha } )}{2} \bigl\vert f'(b) \bigr\vert \biggr]. \end{aligned}$$

### Theorem 6

([17, Corollary 4.14])

*Let* $f: I^{o}\subseteq \mathbb{R}\to \mathbb{R}$ *be a differentiable mapping on* $I^{o}$, $(a,b)\in I^{o}\times I^{o}$, $a< b$. *Suppose that the function* $|f'|$ *is* *h*-*convex on* $[a,b]$. *Then*

$$\begin{aligned} & \biggl\vert \frac{f(a)+f(b)}{2}-\frac{1}{b-a} \int _{a}^{b} f(x)\,dx \biggr\vert \leq (b-a) \biggl( \int _{0}^{1} h(t)\,dt \biggr) \biggl( \frac{ \vert f'(a) \vert + \vert f'(b) \vert }{2} \biggr). \end{aligned}$$

For more recent results on integral inequalities of Hermite–Hadamard type concerning the *F*-convex functions, we refer the interested reader to [19] and the references therein.

In the sequel, we recall the concepts of the left-sided and right-sided Riemann- Liouville fractional integrals of the order $\alpha >0$.

### Definition 2.2

([20])

Suppose that $f\in L([a,b])$. The left and right Riemann–Liouville fractional integrals denoted by $J_{a^{+}}^{\alpha }f$ and $J_{b^{-}}^{\alpha }f$ of order $\alpha >0$ are defined by

$$\begin{aligned} J_{a^{+}}^{\alpha }f(x) &=\frac{1}{\varGamma (\alpha )} \int _{a}^{x} (x-t)^{ \alpha -1}f(t)\,dt,\quad x>a, \end{aligned}$$

and

$$\begin{aligned} J_{b^{-}}^{\alpha }f(x) &=\frac{1}{\varGamma (\alpha )} \int _{x}^{b} (t-x)^{ \alpha -1}f(t)\,dt,\quad x< b, \end{aligned}$$

respectively, where $\varGamma (\alpha )$ is the gamma function defined by $\varGamma (\alpha )=\int _{0}^{\infty }e^{-t}t^{\alpha -1}\,dt$ and $J_{b^{-}}^{0}f(x)=J_{b^{-}}^{0}f(x)=f(x)$.

In [21], authors established the following Hermite–Hadamard type inequalities for *F*-convex functions involving a Riemann–Liouville fractional:

### Theorem 7

*Let* $I\subseteq \mathbb{R}$ *be an interval*, $f: I^{o}\subseteq \mathbb{R}\to \mathbb{R}$ *be a differentiable mapping on* $I^{o}$, $a,b \in I^{o}$, $a< b$. *If* *f* *is* *F*-*convex on* $[a,b]$, *for some* $F\in \mathcal{F}$, *then we have*

$$\begin{aligned} &F \biggl(f \biggl(\frac{a+b}{2} \biggr),\frac{\varGamma (\alpha +1)}{(b-a)^{ \alpha }}J_{a^{+}}^{\alpha }f(b), \frac{\varGamma (\alpha +1)}{(b-a)^{ \alpha }}J_{b^{-}}^{\alpha }f(a),\frac{1}{2} \biggr)+ \int _{0}^{1} L _{w(t)}\,dt\leq 0, \\ &T_{F,w} \biggl(\frac{\varGamma (\alpha +1)}{(b-a)^{\alpha }} \bigl[J _{a^{+}}^{\alpha }f(b)+J_{b^{-}}^{\alpha }f(a) \bigr],f(a)+f(b),f(a)+f(b) \biggr)+ \int _{0}^{1} L_{w(t)}\,dt\leq 0, \end{aligned}$$

*where* $w(t)=\alpha t^{\alpha -1}$.

### Theorem 8

*Let* $I\subseteq \mathbb{R}$ *be an interval*, $f: I^{o}\subseteq \mathbb{R}\to \mathbb{R}$ *be a differentiable mapping on* $I^{o}$, $a,b \in I^{o}$, $a< b$. *If* *f* *is* *F*-*convex on* $[a,b]$, *for some* $F\in \mathcal{F}$ *and the function* $t\in (0,1)\to L_{w(t)}$ *belongs to* $L_{1}(a,b)$, *where* $w(t)= \vert (1-t)^{\alpha }-t^{\alpha } \vert $. *Then we have the inequality*

$$\begin{aligned} &T_{F,w} \biggl(\frac{2}{b-a} \biggl\vert \frac{f(a)+f(b)}{2}- \frac{\varGamma ( \alpha +1)}{2(b-a)^{\alpha }} \bigl[J_{a^{+}}^{\alpha }f(b)+J_{b^{-}} ^{\alpha }f(a) \bigr] \biggr\vert , \bigl\vert f'(a) \bigr\vert , \bigl\vert f'(b) \bigr\vert \biggr)\\ &\quad {}+ \int _{0}^{1} L _{w(t)}\,dt\leq 0. \end{aligned}$$

The following definitions will be useful for this study [20].

### Definition 2.3

The Euler beta function is defined as follows:

$$\begin{aligned} B(a,b)= \int _{0}^{1} t^{a-1}(1-t)^{b-1}\,dt, \quad a,b>0. \end{aligned}$$

The incomplete beta function is defined by

$$\begin{aligned} B_{x}(a,b)= \int _{0}^{x} t^{a-1}(1-t)^{b-1}\,dt, \quad a,b>0. \end{aligned}$$

Note that, for $x=1$, the incomplete beta function reduces to the Euler beta function.

Also, the following three lemmas are important to obtain our main results.

### Lemma 1

([22, Lemma 4])

*Let* $f: [a,b]\to \mathbb{R}$ *be a once differentiable mappings on* $(a,b)$ *with* $a< b$, $\eta (b,a)>0$. *If* $f'\in L [a,a+e^{i \varphi }\eta (b,a) ]$, *then the following equality for the fractional integral holds*:

$$\begin{aligned} &\frac{f(a)+f (a+e^{i \varphi }\eta (b,a) )}{2} -\frac{ \varGamma (\alpha +1))}{2 (e^{i \varphi }\eta (b,a) )^{\alpha }} \bigl[J_{a^{+}}^{\alpha }f \bigl(a+e^{i \varphi }\eta (b,a) \bigr) +J_{ (a+e^{i \varphi }\eta (b,a) )^{-}}^{\alpha }f(a) \bigr] \\ &\quad =\frac{e^{i \varphi }\eta (b,a)}{2} \int _{0}^{1} \bigl[(1-t)^{ \alpha }-t^{\alpha } \bigr] f' \bigl(a+(1-t)e^{i \varphi }\eta (b,a) \bigr)\,dt. \end{aligned}$$

### Lemma 2

([16, Lemma 5])

*Let* $f: [a,b]\to \mathbb{R}$ *be a once differentiable mappings on* $(a,b)$ *with* $a< b$, $\eta (b,a)>0$. *If* $f''\in L [a,a+e^{i \varphi }\eta (b,a) ]$, *then the following equality for the fractional integral holds*:

$$\begin{aligned} &\frac{f(a)+f (a+e^{i \varphi }\eta (b,a) )}{2} -\frac{ \varGamma (\alpha +1))}{2 (e^{i \varphi }\eta (b,a) )^{\alpha }} \bigl[J_{a^{+}}^{\alpha }f \bigl(a+e^{i \varphi }\eta (b,a) \bigr) +J_{ (a+e^{i \varphi }\eta (b,a) )^{-}}^{\alpha }f(a) \bigr] \\ &\quad =\frac{ (e^{i \varphi }\eta (b,a) )^{2}}{2(\alpha +1)} \int _{0}^{1} \bigl[1-(1-t)^{\alpha +1}-t^{\alpha +1} \bigr]f'' \bigl(a+(1-t)e ^{i \varphi }\eta (b,a) \bigr)\,dt. \end{aligned}$$

### Lemma 3

([22])

*For* $t\in [0,1]$, *we have*

$$\begin{aligned} (1-t)^{m} &\leq 2^{1-m}-t^{m}, \quad \textit{for }m \in [0,1], \\ (1-t)^{m} &\geq 2^{1-m}-t^{m}, \quad \textit{for }m \in [1,\infty ). \end{aligned}$$

In this study, using the $\lambda _{\varphi }$-preinvexity of the function, we establish new inequalities of Hermite–Hadamard type for differentiable function and some trapezoid type inequalities for function whose second derivatives absolutely values are *F*-convex.

## Hermite–Hadamard type inequalities for differentiable functions

In this section, we establish some inequalities of Hermite–Hadamard type for *F*-convex functions in fractional integral forms.

### Theorem 9

*Let* $I\subseteq \mathbb{R}$ *be an open invex set with respect to bifunction* $\eta : I\times I\to \mathbb{R}$, *where* $\eta (b,a)>0$. *Let* $f: [0,b]\to \mathbb{R}$ *be a differentiable mapping*. *Suppose that* $|f'|$ *is measurable*, *decreasing*, $\lambda _{\varphi }$-*preinvex function on* *I*, *and* *F*-*convex on* $[a,b]$, *for some* $F\in \mathcal{F}$ *and the function* $t\in (0,1)\to L_{w(t)}$ *belongs to* $L^{1}(0,1)$, *where* $w(t)= \vert (1-t)^{\alpha }-t^{\alpha } \vert $. *Then*

10 $$\begin{aligned} \begin{aligned}[b] &T_{F,w} \biggl(\frac{2}{e^{i \varphi }\eta (b,a)} \biggl\vert \frac{f(a)+f (a+e^{i \varphi } \eta (b,a) )}{2} \\ &\quad {}-\frac{\varGamma (\alpha +1)}{2 (e^{i \varphi }\eta (b,a) )^{\alpha }} \bigl[J_{a^{+}} ^{\alpha }f \bigl(a+e^{i \varphi }\eta (b,a) \bigr) +J_{ (a+e ^{i \varphi }\eta (b,a) )^{-}}^{\alpha }f(a) \bigr] \biggr\vert , \bigl\vert f'(a) \bigr\vert , \bigl\vert f'(b) \bigr\vert \biggr)\\ &\quad {} + \int _{0}^{1} L _{w(t)}\,dt\leq 0. \end{aligned} \end{aligned}$$

### Proof

Since $|f'|$ is *F*-convex, we have

$$\begin{aligned} F \bigl( \bigl\vert f' \bigl(a+(1-t)e^{i \varphi }\eta (b,a) \bigr) \bigr\vert , \bigl\vert f'(a) \bigr\vert , \bigl\vert f'(b) \bigr\vert ,t \bigr)\leq 0,\quad t \in [0,1]. \end{aligned}$$

Multiplying this inequality by $w(t)= \vert (1-t)^{\alpha }-t^{\alpha } \vert $ and using axiom (A3), we have

$$\begin{aligned} F \bigl(w(t) \bigl\vert f' \bigl(a+(1-t)e^{i \varphi }\eta (b,a) \bigr) \bigr\vert ,w(t) \bigl\vert f'(a) \bigr\vert , w(t) \bigl\vert f'(b) \bigr\vert ,t \bigr)+L_{w(t)} \leq 0,\quad t\in [0,1]. \end{aligned}$$

Integrating over $[0,1]$ and using axiom (A2), we get

$$\begin{aligned} &T_{F,w} \biggl( \int _{0}^{1} \bigl\vert (1-t)^{\alpha }-t^{\alpha } \bigr\vert \bigl\vert f' \bigl(a+(1-t)e^{i \varphi } \eta (b,a) \bigr) \bigr\vert \,dt, \bigl\vert f'(a) \bigr\vert , \bigl\vert f'(b) \bigr\vert ,t \biggr)\\ &\quad {}+ \int _{0}^{1} L _{w(t)}\,dt \leq 0,\quad t\in [0,1]. \end{aligned}$$

But from Lemma 1 we have

$$\begin{aligned} &\frac{2}{e^{i \varphi }\eta (b,a)} \biggl\vert \frac{f(a)+f (a+e^{i \varphi }\eta (b,a) )}{2} \\ &\qquad {}-\frac{\varGamma (\alpha +1))}{2 (e ^{i \varphi }\eta (b,a) )^{\alpha }} \bigl[J_{a^{+}}^{\alpha }f \bigl(a+e^{i \varphi }\eta (b,a) \bigr) +J_{ (a+e^{i \varphi } \eta (b,a) )^{-}}^{\alpha }f(a) \bigr] \biggr\vert \\ &\quad \leq \int _{0}^{1} \bigl\vert (1-t)^{\alpha }-t^{\alpha } \bigr\vert \bigl\vert f' \bigl(a+(1-t)e^{i \varphi } \eta (b,a) \bigr) \bigr\vert \,dt. \end{aligned}$$

Because $T_{F,w}$ is nondecreasing with respect to the first variable so that

$$\begin{aligned} &T_{F,w} \biggl(\frac{2}{e^{i \varphi }\eta (b,a)} \biggl\vert \frac{f(a)+f (a+e^{i \varphi } \eta (b,a) )}{2} \\ &\quad {}- \frac{\varGamma (\alpha +1)}{2 (e^{i \varphi }\eta (b,a) )^{\alpha }} \bigl[J_{a^{+}} ^{\alpha }f \bigl(a+e^{i \varphi } \eta (b,a) \bigr) +J_{ (a+e ^{i \varphi }\eta (b,a) )^{-}}^{\alpha }f(a) \bigr] \biggr\vert , \bigl\vert f'(a) \bigr\vert , \bigl\vert f'(b) \bigr\vert \biggr) \\ &\quad {}+ \int _{0}^{1} L _{w(t)}\,dt\leq 0,\quad t\in [0,1]. \end{aligned}$$

This proves (10). □

### Remark 2

If we choose $\eta (b,a)=b-a$ and $\varphi =0$ in Theorem 9, we get

$$\begin{aligned} &T_{F,w} \biggl(\frac{2}{b-a} \biggl\vert \frac{f(a)+f(b)}{2}- \frac{\varGamma ( \alpha +1))}{2(b-a)^{\alpha }} \bigl[J_{a^{+}}^{\alpha }f(b)+J_{b ^{-}}^{\alpha }f(a) \bigr] \biggr\vert , \bigl\vert f'(a) \bigr\vert , \bigl\vert f'(b) \bigr\vert \biggr)\\ &\quad {} + \int _{0}^{1} L_{w(t)}\,dt\leq 0. \end{aligned}$$

### Corollary 1

*Under the assumptions of Theorem *9, *if* $|f'|$ *is* *ε*-*convex*, *then we have*

$$\begin{aligned} & \biggl\vert \frac{f(a)+f (a+e^{i \varphi } \eta (b,a) )}{2}-\frac{ \varGamma (\alpha +1))}{2 (e^{i \varphi }\eta (b,a) )^{\alpha }} \bigl[J_{a^{+}}^{\alpha }f \bigl(a+e^{i \varphi }\eta (b,a) \bigr) +J_{ (a+e^{i \varphi }\eta (b,a) )^{-}}^{\alpha }f(a) \bigr] \biggr\vert \\ &\quad \leq \frac{e^{i \varphi }\eta (b,a)}{2(\alpha +1)} \biggl(1-\frac{1}{2^{ \alpha }} \biggr) \bigl( \bigl\vert f'(a) \bigr\vert + \bigl\vert f'(b) \bigr\vert +2 \varepsilon \bigr). \end{aligned}$$

### Proof

Using (5) with $w(t)= \vert (1-t)^{\alpha }-t^{\alpha } \vert $, we find

$$\begin{aligned} \int _{0}^{1} L_{w(t)}\,dt &=\varepsilon \int _{0}^{1} \bigl(1- \bigl\vert (1-t)^{ \alpha }-t^{\alpha } \bigr\vert \bigr)\,dt \\ &=\varepsilon \biggl[ \int _{0}^{\frac{1}{2}} \bigl(1-(1-t)^{\alpha }+t ^{\alpha } \bigr)\,dt + \int _{1}^{\frac{1}{2}} \bigl(1-(1-t)^{\alpha }+t ^{\alpha } \bigr)\,dt \biggr] \\ &=\varepsilon \biggl[1-\frac{2}{\alpha +1} \biggl(1-\frac{1}{\alpha } \biggr) \biggr]. \end{aligned}$$

From (4) with $w(t)= \vert (1-t)^{\alpha }-t^{\alpha } \vert $, we have

$$\begin{aligned} &T_{F,w}(u_{1},u_{2},u_{3}) \\ &\quad =u_{1}- \biggl( \int _{0}^{1} t \bigl\vert (1-t)^{\alpha }-t^{\alpha } \bigr\vert \,dt \biggr)u _{2} - \biggl( \int _{0}^{1} (1-t) \bigl\vert (1-t)^{\alpha }-t^{\alpha } \bigr\vert \,dt \biggr)u _{3}- \varepsilon \\ &\quad =u_{1}-\frac{1}{\alpha +1} \biggl(1-\frac{1}{\alpha } \biggr) (u_{2}+u _{3})-\varepsilon , \end{aligned}$$

for $u_{1}, u_{2}, u_{3}\in \mathbb{R}$. Hence, by Theorem 9, we have

$$\begin{aligned} 0 &\geq T_{F,w} \biggl(\frac{2}{e^{i \varphi }\eta (b,a)} \biggl\vert \frac{f(a)+f (a+e^{i \varphi } \eta (b,a) )}{2} \\ &\quad {}-\frac{\varGamma (\alpha +1)}{2 (e^{i \varphi }\eta (b,a) )^{\alpha }} \bigl[J_{a^{+}} ^{\alpha }f \bigl(a+e^{i \varphi }\eta (b,a) \bigr) +J_{ (a+e ^{i \varphi }\eta (b,a) )^{-}}^{\alpha }f(a) \bigr] \biggr\vert , \bigl\vert f'(a) \bigr\vert , \bigl\vert f'(b) \bigr\vert \biggr)\\ &\quad {}+ \int _{0}^{1} L_{w(t)}\,dt \\ &=\frac{2}{e^{i \varphi }\eta (b,a)} \biggl\vert \frac{f(a)+f (a+e ^{i \varphi } \eta (b,a) )}{2}\\ &\quad {}-\frac{\varGamma (\alpha +1)}{2 (e ^{i \varphi }\eta (b,a) )^{\alpha }} \bigl[J_{a^{+}}^{\alpha }f \bigl(a+e^{i \varphi }\eta (b,a) \bigr) +J_{ (a+e^{i \varphi } \eta (b,a) )^{-}}^{\alpha }f(a) \bigr] \biggr\vert \\ &\quad{}-\frac{1}{\alpha +1} \biggl(1-\frac{1}{\alpha } \biggr) \bigl( \bigl\vert f'(a) \bigr\vert + \bigl\vert f'(b) \bigr\vert +2\varepsilon \bigr). \end{aligned}$$

This completes the proof. □

### Remark 3

In Corollary 1, if we choose

- $\eta (b,a)=b-a$ and $\varphi =0$, we get
  $$\begin{aligned} & \biggl\vert \frac{f(a)+f(b)}{2}-\frac{\varGamma (\alpha +1))}{2(b-a)^{\alpha }} \bigl[J_{a^{+}}^{\alpha }f(b)+J_{b^{-}}^{\alpha }f(a) \bigr] \biggr\vert \\ &\quad \leq \frac{b-a}{2(\alpha +1)} \biggl(1-\frac{1}{2^{\alpha }} \biggr) \bigl( \bigl\vert f'(a) \bigr\vert + \bigl\vert f'(b) \bigr\vert +2\varepsilon \bigr). \end{aligned}$$
- $\eta (b,a)=b-a$, $\varphi =0$, and $\varepsilon =0$, we get
  $$\begin{aligned} & \biggl\vert \frac{f(a)+f(b)}{2}-\frac{\varGamma (\alpha +1))}{2(b-a)^{\alpha }} \bigl[J_{a^{+}}^{\alpha }f(b)+J_{b^{-}}^{\alpha }f(a) \bigr] \biggr\vert \\ &\quad \leq \frac{b-a}{2(\alpha +1)} \biggl(1-\frac{1}{2^{\alpha }} \biggr) \bigl( \bigl\vert f'(a) \bigr\vert + \bigl\vert f'(b) \bigr\vert \bigr) \end{aligned}$$
  which is given by [18].

### Corollary 2

*Under the assumptions of Theorem *9, *if* $|f'|$ *is* $\lambda _{\varphi }$-*preinvex*, *then we have*

$$\begin{aligned} & \biggl\vert \frac{f(a)+f (a+e^{i \varphi } \eta (b,a) )}{2}-\frac{ \varGamma (\alpha +1))}{2 (e^{i \varphi }\eta (b,a) )^{\alpha }} \bigl[J_{a^{+}}^{\alpha }f \bigl(a+e^{i \varphi }\eta (b,a) \bigr) +J_{ (a+e^{i \varphi }\eta (b,a) )^{-}}^{\alpha }f(a) \bigr] \biggr\vert \\ &\quad \leq \frac{e^{i \varphi }\eta (b,a)}{4} \biggl[B_{\frac{1}{2}} \biggl(\frac{1}{2}, \alpha +\frac{1}{2} \biggr) -B_{\frac{1}{2}} \biggl(\alpha + \frac{1}{2},\frac{1}{2} \biggr) \biggr] \biggl( \bigl\vert f'(a) \bigr\vert +\frac{1- \lambda }{\lambda } \bigl\vert f'(b) \bigr\vert \biggr). \end{aligned}$$

### Proof

Using (7) with $w(t)= \vert (1-t)^{\alpha }-t^{\alpha } \vert $, we have

$$\begin{aligned} &T_{F,w}(u_{1},u_{2},u_{3}) \\ &\quad =u_{1}- \biggl( \int _{0}^{1} \frac{\sqrt{t}}{2\sqrt{1-t}} \bigl\vert (1-t)^{ \alpha }-t^{\alpha } \bigr\vert \,dt \biggr)u_{3}- \frac{1-\lambda }{\lambda } \biggl( \int _{0}^{1} \frac{\sqrt{1-t}}{2\sqrt{t}} \bigl\vert (1-t)^{ \alpha }-t^{\alpha } \bigr\vert \,dt \biggr)u_{2} \\ &\quad =u_{1}-\frac{1}{2} \biggl[B_{\frac{1}{2}} \biggl( \frac{1}{2},\alpha + \frac{1}{2} \biggr) -B_{\frac{1}{2}} \biggl( \alpha +\frac{1}{2}, \frac{1}{2} \biggr) \biggr] \biggl(u_{2}+ \frac{1-\lambda }{\lambda }u _{3} \biggr) \end{aligned}$$

for $u_{1}, u_{2}, u_{3}\in \mathbb{R}$. Hence, by Theorem 9, we have

$$\begin{aligned} 0 &\geq T_{F,w} \biggl(\frac{2}{e^{i \varphi }\eta (b,a)} \biggl\vert \frac{f(a)+f (a+e^{i \varphi } \eta (b,a) )}{2} \\ &\quad {}-\frac{\varGamma (\alpha +1)}{2 (e^{i \varphi }\eta (b,a) )^{\alpha }} \bigl[J_{a^{+}} ^{\alpha }f \bigl(a+e^{i \varphi }\eta (b,a) \bigr) +J_{ (a+e ^{i \varphi }\eta (b,a) )^{-}}^{\alpha }f(a) \bigr] \biggr\vert , \bigl\vert f'(a) \bigr\vert , \bigl\vert f'(b) \bigr\vert \biggr)\\ &\quad {}+ \int _{0}^{1} L_{w(t)}\,dt \\ &=\frac{2}{e^{i \varphi }\eta (b,a)} \biggl\vert \frac{f(a)+f (a+e ^{i \varphi } \eta (b,a) )}{2}\\ &\quad {}-\frac{\varGamma (\alpha +1)}{2 (e ^{i \varphi }\eta (b,a) )^{\alpha }} \bigl[J_{a^{+}}^{\alpha }f \bigl(a+e^{i \varphi }\eta (b,a) \bigr) +J_{ (a+e^{i \varphi } \eta (b,a) )^{-}}^{\alpha }f(a) \bigr] \biggr\vert \\ &\quad{}-\frac{1}{2} \biggl[B_{\frac{1}{2}} \biggl(\frac{1}{2}, \alpha + \frac{1}{2} \biggr) -B_{\frac{1}{2}} \biggl(\alpha + \frac{1}{2}, \frac{1}{2} \biggr) \biggr] \biggl( \bigl\vert f'(a) \bigr\vert +\frac{1- \lambda }{\lambda } \bigl\vert f'(b) \bigr\vert \biggr). \end{aligned}$$

This leads to

$$\begin{aligned} & \biggl\vert \frac{f(a)+f (a+e^{i \varphi } \eta (b,a) )}{2}-\frac{ \varGamma (\alpha +1)}{2 (e^{i \varphi }\eta (b,a) )^{\alpha }} \bigl[J_{a^{+}}^{\alpha }f \bigl(a+e^{i \varphi }\eta (b,a) \bigr) +J_{ (a+e^{i \varphi }\eta (b,a) )^{-}}^{\alpha }f(a) \bigr] \biggr\vert \\ &\quad \leq \frac{e^{i \varphi }\eta (b,a)}{4} \biggl[B_{\frac{1}{2}} \biggl(\frac{1}{2}, \alpha +\frac{1}{2} \biggr) -B_{\frac{1}{2}} \biggl(\alpha + \frac{1}{2},\frac{1}{2} \biggr) \biggr] \biggl( \bigl\vert f'(a) \bigr\vert +\frac{1- \lambda }{\lambda } \bigl\vert f'(b) \bigr\vert \biggr). \end{aligned}$$

Thus, the proof is done. □

### Remark 4

In Corollary 2, if we choose

- $\eta (b,a)=b-a$ and $\varphi =0$, we get
  $$\begin{aligned} & \biggl\vert \frac{f(a)+f(b)}{2}-\frac{\varGamma (\alpha +1))}{2(b-a)^{\alpha }} \bigl[J_{a^{+}}^{\alpha }f(b)+J_{b^{-}}^{\alpha }f(a) \bigr] \biggr\vert \\ &\quad \leq \frac{b-a}{4} \biggl[B_{\frac{1}{2}} \biggl(\frac{1}{2}, \alpha + \frac{1}{2} \biggr) -B_{\frac{1}{2}} \biggl(\alpha + \frac{1}{2}, \frac{1}{2} \biggr) \biggr] \biggl( \bigl\vert f'(a) \bigr\vert +\frac{1- \lambda }{\lambda } \bigl\vert f'(b) \bigr\vert \biggr). \end{aligned}$$
- $\eta (b,a)=b-a$, $\varphi =0$, and $\lambda =\frac{1}{2}$, we get
  $$\begin{aligned} & \biggl\vert \frac{f(a)+f(b)}{2}-\frac{\varGamma (\alpha +1))}{2(b-a)^{\alpha }} \bigl[J_{a^{+}}^{\alpha }f(b)+J_{b^{-}}^{\alpha }f(a) \bigr] \biggr\vert \\ &\quad \leq \frac{b-a}{4} \biggl[B_{\frac{1}{2}} \biggl( \frac{1}{2},\alpha + \frac{1}{2} \biggr) -B_{\frac{1}{2}} \biggl( \alpha +\frac{1}{2}, \frac{1}{2} \biggr) \biggr] \bigl( \bigl\vert f'(a) \bigr\vert + \bigl\vert f'(b) \bigr\vert \bigr). \end{aligned}$$

### Corollary 3

*Under the assumptions of Theorem *9, *if* $|f'|$ *is* *h*-*convex*, *then we have*

$$\begin{aligned} & \biggl\vert \frac{f(a)+f (a+e^{i \varphi } \eta (b,a) )}{2}-\frac{ \varGamma (\alpha +1))}{2 (e^{i \varphi }\eta (b,a) )^{\alpha }} \bigl[J_{a^{+}}^{\alpha }f \bigl(a+e^{i \varphi }\eta (b,a) \bigr) +J_{ (a+e^{i \varphi }\eta (b,a) )^{-}}^{\alpha }f(a) \bigr] \biggr\vert \\ &\quad \leq \frac{e^{i \varphi }\eta (b,a)}{2} \biggl( \int _{0}^{1} h(t) \bigl\vert (1-t)^{\alpha }-t^{\alpha } \bigr\vert \,dt \biggr) \biggl( \bigl\vert f'(a) \bigr\vert + \frac{1- \lambda }{\lambda } \bigl\vert f'(b) \bigr\vert \biggr). \end{aligned}$$

### Proof

Using (9) with $w(t)= \vert (1-t)^{\alpha }-t^{\alpha } \vert $, we have

$$\begin{aligned} &T_{F,w}(u_{1},u_{2},u_{3}) \\ &\quad =u_{1}- \biggl( \int _{0}^{1} h(t) \bigl\vert (1-t)^{\alpha }-t^{\alpha } \bigr\vert \,dt \biggr)u _{3} -\frac{1-\lambda }{\lambda } \biggl( \int _{0}^{1} h(1-t) \bigl\vert (1-t)^{ \alpha }-t^{\alpha } \bigr\vert \,dt \biggr)u_{2} \\ &\quad =u_{1}- \biggl( \int _{0}^{1} h(t) \bigl\vert (1-t)^{\alpha }-t^{\alpha } \bigr\vert \,dt \biggr)u _{3} -\frac{1-\lambda }{\lambda } \biggl( \int _{0}^{1} h(t) \bigl\vert (1-t)^{ \alpha }-t^{\alpha } \bigr\vert \,dt \biggr)u_{2} \\ &\quad =u_{1}- \biggl( \int _{0}^{1} h(t) \bigl\vert (1-t)^{\alpha }-t^{\alpha } \bigr\vert \,dt \biggr) \biggl(u_{2}+\frac{1- \lambda }{\lambda }u_{3} \biggr) \end{aligned}$$

for $u_{1}, u_{2}, u_{3}\in \mathbb{R}$. So, by Theorem 9, we have

$$\begin{aligned} 0 &\geq T_{F,w} \biggl(\frac{2}{e^{i \varphi }\eta (b,a)} \biggl\vert \frac{f(a)+f (a+e^{i \varphi } \eta (b,a) )}{2}\\ &\quad {}-\frac{\varGamma (\alpha +1)}{2 (e^{i \varphi }\eta (b,a) )^{\alpha }} \bigl[J_{a^{+}} ^{\alpha }f \bigl(a+e^{i \varphi }\eta (b,a) \bigr) +J_{ (a+e ^{i \varphi }\eta (b,a) )^{-}}^{\alpha }f(a) \bigr] \biggr\vert ,\bigl\vert f'(a) \bigr\vert , \bigl\vert f'(b) \bigr\vert \biggr) \\ & \quad {}+ \int _{0}^{1} L_{w(t)}\,dt \\ &=\frac{2}{e^{i \varphi }\eta (b,a)} \biggl\vert \frac{f(a)+f (a+e ^{i \varphi } \eta (b,a) )}{2}\\ &\quad {}-\frac{\varGamma (\alpha +1)}{2 (e ^{i \varphi }\eta (b,a) )^{\alpha }} \bigl[J_{a^{+}}^{\alpha }f \bigl(a+e^{i \varphi }\eta (b,a) \bigr) +J_{ (a+e^{i \varphi } \eta (b,a) )^{-}}^{\alpha }f(a) \bigr] \biggr\vert \\ &\quad {}- \biggl( \int _{0}^{1} h(t) \bigl\vert (1-t)^{\alpha }-t^{\alpha } \bigr\vert \,dt \biggr) \biggl( \bigl\vert f'(a) \bigr\vert + \frac{1- \lambda }{\lambda } \bigl\vert f'(b) \bigr\vert \biggr). \end{aligned}$$

This leads to

$$\begin{aligned} & \biggl\vert \frac{f(a)+f (a+e^{i \varphi } \eta (b,a) )}{2}-\frac{ \varGamma (\alpha +1)}{2 (e^{i \varphi }\eta (b,a) )^{\alpha }} \bigl[J_{a^{+}}^{\alpha }f \bigl(a+e^{i \varphi }\eta (b,a) \bigr) +J_{ (a+e^{i \varphi }\eta (b,a) )^{-}}^{\alpha }f(a) \bigr] \biggr\vert \\ &\quad \leq \frac{e^{i \varphi }\eta (b,a)}{2} \biggl( \int _{0}^{1} h(t) \bigl\vert (1-t)^{\alpha }-t^{\alpha } \bigr\vert \,dt \biggr) \biggl( \bigl\vert f'(a) \bigr\vert + \frac{1- \lambda }{\lambda } \bigl\vert f'(b) \bigr\vert \biggr). \end{aligned}$$

Thus, the proof is done. □

### Theorem 10

*Let* $I\subseteq \mathbb{R}$ *be an open invex set with respect to bifunction* $\eta : I\times I\to \mathbb{R}$, *where* $\eta (b,a)>0$. *Let* $f: [0,b]\to \mathbb{R}$ *be a differentiable mapping*. *Suppose that* $|f'|^{{\frac{p}{p-1}}}$ *is measurable*, *decreasing*, $\lambda _{\varphi }$-*preinvex function on* *I*, *and* *F*-*convex on* $[a,b]$, *for some* $F\in \mathcal{F}$ *and* $|f'|\in L^{{\frac{p}{p-1}}}(a,b)$. *Then*

11 $$\begin{aligned} &T_{F,1} \bigl(G_{1}(f,p), \bigl\vert f'(a) \bigr\vert ^{\frac{p}{p-1}}, \bigl\vert f'(b) \bigr\vert ^{\frac{p}{p-1}} \bigr)\leq 0, \end{aligned}$$

*where*

$$\begin{aligned} G_{1}(f,p) &= \biggl(\frac{2}{e^{i \varphi }\eta (b,a)} \biggr)^{ \frac{p}{p-1}} \biggl( \frac{\alpha p+1}{2-2^{1-\alpha p}} \biggr)^{ \frac{1}{p-1}} \biggl\vert \frac{f(a)+f (a+e^{i \varphi } \eta (b,a) )}{2} \\ &\quad {}-\frac{ \varGamma (\alpha +1)}{2 (e^{i \varphi }\eta (b,a) )^{\alpha }} \bigl[J_{a^{+}}^{\alpha }f \bigl(a+e^{i \varphi }\eta (b,a) \bigr) +J_{ (a+e^{i \varphi }\eta (b,a) )^{-}}^{\alpha }f(a) \bigr] \biggr\vert ^{\frac{p}{p-1}}. \end{aligned}$$

### Proof

Since $|f'|^{\frac{p}{p-1}}$ is *F*-convex, we have

$$\begin{aligned} F \bigl( \bigl\vert f' \bigl(a+(1-t)e^{i \varphi }\eta (b,a) \bigr) \bigr\vert ^{\frac{p}{p-1}}, \bigl\vert f'(a) \bigr\vert ^{\frac{p}{p-1}}, \bigl\vert f'(b) \bigr\vert ^{\frac{p}{p-1}},t \bigr) \leq 0,\quad t\in [0,1]. \end{aligned}$$

With $w(t)=1$ in (A2), we have

$$\begin{aligned} T_{F,1} \biggl( \int _{0}^{1} \bigl\vert f' \bigl(a+(1-t)e^{i \varphi }\eta (b,a) \bigr) \bigr\vert ^{\frac{p}{p-1}}\,dt, \bigl\vert f'(a) \bigr\vert ^{\frac{p}{p-1}}, \bigl\vert f'(b) \bigr\vert ^{\frac{p}{p-1}} \biggr)\leq 0, \quad t\in [0,1]. \end{aligned}$$

Using Lemma 1 and the Hölder inequality, we get

$$\begin{aligned} & \biggl\vert \frac{f(a)+f (a+e^{i \varphi } \eta (b,a) )}{2}-\frac{ \varGamma (\alpha +1)}{2 (e^{i \varphi }\eta (b,a) )^{\alpha }} \bigl[J_{a^{+}}^{\alpha }f \bigl(a+e^{i \varphi }\eta (b,a) \bigr) +J_{ (a+e^{i \varphi }\eta (b,a) )^{-}}^{\alpha }f(a) \bigr] \biggr\vert \\ &\quad =\frac{e^{i \varphi }\eta (b,a)}{2} \int _{0}^{1} \bigl\vert (1-t)^{ \alpha }-t^{\alpha } \bigr\vert \bigl\vert f' \bigl(a+(1-t)e^{i \varphi } \eta (b,a) \bigr) \bigr\vert \,dt \\ &\quad \leq \frac{e^{i \varphi }\eta (b,a)}{2} \biggl( \int _{0}^{1} \bigl\vert (1-t)^{ \alpha }-t^{\alpha } \bigr\vert \,dt \biggr)^{\frac{1}{p}} \biggl( \int _{0} ^{1} \bigl\vert f' \bigl(a+(1-t)e^{i \varphi }\eta (b,a) \bigr) \bigr\vert ^{\frac{p}{p-1}}\,dt \biggr)^{\frac{p-1}{p}} \\ &\quad = \frac{e^{i \varphi }\eta (b,a)}{2} \biggl(\frac{2-2^{1-\alpha p}}{ \alpha p+1} \biggr)^{\frac{1}{p}} \biggl( \int _{0}^{1} \bigl\vert f' \bigl(a+(1-t)e ^{i \varphi }\eta (b,a) \bigr) \bigr\vert ^{\frac{p}{p-1}}\,dt \biggr) ^{\frac{p-1}{p}} \end{aligned}$$

or, equivalently,

$$\begin{aligned} & \biggl(\frac{2}{e^{i \varphi }\eta (b,a)} \biggr)^{\frac{p}{p-1}} \biggl(\frac{\alpha p+1}{2-2^{1-\alpha p}} \biggr)^{\frac{1}{p-1}} \biggl\vert \frac{f(a)+f (a+e^{i \varphi } \eta (b,a) )}{2} \\ &\qquad {}-\frac{ \varGamma (\alpha +1)}{2 (e^{i \varphi }\eta (b,a) )^{\alpha }} \bigl[J_{a^{+}}^{\alpha }f \bigl(a+e^{i \varphi }\eta (b,a) \bigr) +J_{ (a+e^{i \varphi }\eta (b,a) )^{-}}^{\alpha }f(a) \bigr] \biggr\vert ^{\frac{p}{p-1}} \\ &\quad \leq \int _{0}^{1} \bigl\vert f' \bigl(a+(1-t)e^{i \varphi }\eta (b,a) \bigr) \bigr\vert ^{\frac{p}{p-1}}\,dt. \end{aligned}$$

Because $T_{F,1}$ is nondecreasing with respect to the first variable, we get

$$\begin{aligned} &T_{F,1} \bigl(G_{1}(f,p), \bigl\vert f'(a) \bigr\vert ^{{\frac{p}{p-1}}}, \bigl\vert f'(b) \bigr\vert ^{{\frac{p}{p-1}}} \bigr)\leq 0. \end{aligned}$$

Thus, the proof is completed. □

### Remark 5

If we choose $\eta (b,a)=b-a$ and $\varphi =0$ in Theorem 10, we get

$$\begin{aligned} &T_{F,1} \biggl( \biggl(\frac{2}{b-a} \biggr)^{\frac{p}{p-1}} \biggl(\frac{ \alpha p+1}{2-2^{1-\alpha p}} \biggr)^{{\frac{1}{p-1}}} \biggl\vert \frac{f(a)+f(b)}{2} - \frac{\varGamma (\alpha +1))}{2(b-a)^{\alpha }} \bigl[J_{a^{+}}^{\alpha }f(b)+J_{b^{-}}^{\alpha }f(a) \bigr] \biggr\vert ,\\ &\quad \bigl\vert f'(a) \bigr\vert , \bigl\vert f'(b) \bigr\vert \biggr)\leq 0. \end{aligned}$$

### Corollary 4

*Under the assumptions of Theorem *10, *if* $|f'|^{\frac{p}{p-1}}$ *is* *ε*-*convex*, *we have*

$$\begin{aligned} & \biggl\vert \frac{f(a)+f (a+e^{i \varphi } \eta (b,a) )}{2}-\frac{ \varGamma (\alpha +1))}{2 (e^{i \varphi }\eta (b,a) )^{\alpha }} \bigl[J_{a^{+}}^{\alpha }f \bigl(a+e^{i \varphi }\eta (b,a) \bigr) +J_{ (a+e^{i \varphi }\eta (b,a) )^{-}}^{\alpha }f(a) \bigr] \biggr\vert \\ &\quad \leq \frac{e^{i \varphi }\eta (b,a)}{2} \biggl(\frac{2-2^{1-\alpha p}}{ \alpha p+1} \biggr)^{\frac{1}{p}} \biggl(\frac{ \vert f'(a) \vert ^{\frac{p}{p-1}}+ \vert f'(b) \vert ^{\frac{p}{p-1}}}{2} +\varepsilon \biggr)^{\frac{p-1}{p}}. \end{aligned}$$

### Proof

Using (5) with $w(t)=1$, we have

12 $$\begin{aligned} \int _{0}^{1} L_{w(t)}\,dt &=\varepsilon \int _{0}^{1} \bigl(1-w(t)\bigr)\,dt=0. \end{aligned}$$

From (4) with $w(t)=1$, we have

13 $$\begin{aligned} &T_{F,1}(u_{1},u_{2},u_{3}) \\ &\quad =u_{1}- \biggl( \int _{0}^{1} t\,dt \biggr)u_{2}- \biggl( \int _{0}^{1} (1-t)\,dt \biggr)u _{3}- \varepsilon \\ &\quad =u_{1}-\frac{u_{2}+u_{3}}{2}-\varepsilon , \end{aligned}$$

for $u_{1}, u_{2}, u_{3}\in \mathbb{R}$. Hence, by Theorem 10, we have

$$\begin{aligned} 0 &\geq T_{F,1} \bigl(G_{1}(f,p), \bigl\vert f'(a) \bigr\vert ^{\frac{p}{p-1}}, \bigl\vert f'(b) \bigr\vert ^{\frac{p}{p-1}} \bigr) \\ &=G_{1}(f,p)-\frac{ \vert f'(a) \vert ^{\frac{p}{p-1}}+ \vert f'(b) \vert ^{\frac{p}{p-1}}}{2} -\varepsilon . \end{aligned}$$

This leads to

$$\begin{aligned} & \biggl(\frac{2}{e^{i \varphi }\eta (b,a)} \biggr)^{\frac{p}{p-1}} \biggl(\frac{\alpha p+1}{2-2^{1-\alpha p}} \biggr)^{\frac{1}{p-1}} \biggl\vert \frac{f(a)+f (a+e^{i \varphi } \eta (b,a) )}{2} \\ &\quad {}-\frac{ \varGamma (\alpha +1)}{2 (e^{i \varphi }\eta (b,a) )^{\alpha }} \bigl[J_{a^{+}}^{\alpha }f \bigl(a+e^{i \varphi }\eta (b,a) \bigr) +J_{ (a+e^{i \varphi }\eta (b,a) )^{-}}^{\alpha }f(a) \bigr] \biggr\vert ^{\frac{p}{p-1}} \\ &\quad {}-\frac{ \vert f'(a) \vert ^{\frac{p}{p-1}}+ \vert f'(b) \vert ^{\frac{p}{p-1}}}{2} \leq 0 \end{aligned}$$

or, equivalently,

$$\begin{aligned} & \biggl\vert \frac{f(a)+f (a+e^{i \varphi } \eta (b,a) )}{2}-\frac{ \varGamma (\alpha +1))}{2 (e^{i \varphi }\eta (b,a) )^{\alpha }} \bigl[J_{a^{+}}^{\alpha }f \bigl(a+e^{i \varphi }\eta (b,a) \bigr) +J_{ (a+e^{i \varphi }\eta (b,a) )^{-}}^{\alpha }f(a) \bigr] \biggr\vert \\ &\quad \leq \frac{e^{i \varphi }\eta (b,a)}{2} \biggl(\frac{2-2^{1-\alpha p}}{ \alpha p+1} \biggr)^{\frac{1}{p}} \biggl(\frac{ \vert f'(a) \vert ^{\frac{p}{p-1}}+ \vert f'(b) \vert ^{\frac{p}{p-1}}}{2} +\varepsilon \biggr)^{\frac{p-1}{p}}. \end{aligned}$$

This completes the proof. □

### Remark 6

In Corollary 4, if we choose

- $\eta (b,a)=b-a$ and $\varphi =0$, we get
  $$\begin{aligned} & \biggl\vert \frac{f(a)+f(b)}{2}-\frac{\varGamma (\alpha +1))}{2(b-a)^{\alpha }} \bigl[J_{a^{+}}^{\alpha }f(b)+J_{b^{-}}^{\alpha }f(a) \bigr] \biggr\vert \\ &\quad \leq \frac{b-a}{2} \biggl(\frac{2-2^{1-\alpha p}}{\alpha p+1} \biggr) ^{\frac{1}{p}} \biggl(\frac{ \vert f'(a) \vert ^{\frac{p}{p-1}}+ \vert f'(b) \vert ^{\frac{p}{p-1}}}{2}+\varepsilon \biggr)^{\frac{p-1}{p}}. \end{aligned}$$
- $\eta (b,a)=b-a$, $\varphi =0$, and $\varepsilon =0$, we get
  $$\begin{aligned} & \biggl\vert \frac{f(a)+f(b)}{2}-\frac{\varGamma (\alpha +1))}{2(b-a)^{\alpha }} \bigl[J_{a^{+}}^{\alpha }f(b)+J_{b^{-}}^{\alpha }f(a) \bigr] \biggr\vert \\ &\quad \leq \frac{b-a}{2} \biggl(\frac{2-2^{1-\alpha p}}{\alpha p+1} \biggr) ^{\frac{1}{p}} \biggl(\frac{ \vert f'(a) \vert ^{\frac{p}{p-1}}+ \vert f'(b) \vert ^{\frac{p}{p-1}}}{2} \biggr)^{\frac{p-1}{p}}. \end{aligned}$$

### Corollary 5

*Under the assumptions of Theorem *10. *If* $|f'|^{\frac{p-1}{p}}$ *is* $\lambda _{\varphi }$-*preinvex*, *we have*

$$\begin{aligned} & \biggl\vert \frac{f(a)+f (a+e^{i \varphi } \eta (b,a) )}{2}-\frac{ \varGamma (\alpha +1))}{2 (e^{i \varphi }\eta (b,a) )^{\alpha }} \bigl[J_{a^{+}}^{\alpha }f \bigl(a+e^{i \varphi }\eta (b,a) \bigr) +J_{ (a+e^{i \varphi }\eta (b,a) )^{-}}^{\alpha }f(a) \bigr] \biggr\vert \\ &\quad \leq \frac{e^{i \varphi }\eta (b,a)}{2} \biggl(\frac{2-2^{1-\alpha p}}{ \alpha p+1} \biggr)^{\frac{1}{p}} \biggl[\frac{\pi }{4} \biggl( \bigl\vert f'(a) \bigr\vert ^{\frac{p-1}{p}} +\frac{1-\lambda }{\lambda } \bigl\vert f'(b) \bigr\vert ^{ \frac{p-1}{p}} \biggr) \biggr]^{\frac{p-1}{p}}. \end{aligned}$$

### Proof

Using (7) with $w(t)=1$, we have

14 $$\begin{aligned} &T_{F,1}(u_{1},u_{2},u_{3}) \\ &\quad =u_{1}- \biggl( \int _{0}^{1} \frac{\sqrt{t}}{2\sqrt{1-t}}\,dt \biggr)u _{3}-\frac{1-\lambda }{\lambda } \biggl( \int _{0}^{1} \frac{ \sqrt{1-t}}{2\sqrt{t}}\,dt \biggr)u_{2} \\ &\quad =u_{1}-\frac{1}{2}\beta \biggl(\frac{1}{2}, \frac{3}{2} \biggr) \biggl(u_{2}+\frac{1-\lambda }{\lambda }u_{3} \biggr) =u_{1}-\frac{ \pi }{4} \biggl(u_{2}+ \frac{1-\lambda }{\lambda }u_{3} \biggr) \end{aligned}$$

for $u_{1}, u_{2}, u_{3}\in \mathbb{R}$. So, by Theorem 10, we have

$$\begin{aligned} 0 &\geq T_{F,1} \bigl(G_{1}(f,p), \bigl\vert f'(a) \bigr\vert ^{\frac{p-1}{p}}, \bigl\vert f'(b) \bigr\vert ^{\frac{p-1}{p}} \bigr) \\ &= \biggl(\frac{2}{e^{i \varphi }\eta (b,a)} \biggr)^{\frac{p}{p-1}} \biggl(\frac{\alpha p+1}{2-2^{1-\alpha p}} \biggr)^{\frac{1}{p-1}} \biggl\vert \frac{f(a)+f (a+e^{i \varphi } \eta (b,a) )}{2} \\ &\quad {}-\frac{ \varGamma (\alpha +1)}{2 (e^{i \varphi }\eta (b,a) )^{\alpha }} \bigl[J_{a^{+}}^{\alpha }f \bigl(a+e^{i \varphi }\eta (b,a) \bigr) +J_{ (a+e^{i \varphi }\eta (b,a) )^{-}}^{\alpha }f(a) \bigr] \biggr\vert ^{\frac{p}{p-1}} \\ &\quad {}-\frac{\pi }{4} \biggl( \bigl\vert f'(a) \bigr\vert ^{\frac{p-1}{p}} +\frac{1- \lambda }{\lambda } \bigl\vert f'(b) \bigr\vert ^{\frac{p-1}{p}} \biggr). \end{aligned}$$

This leads to

$$\begin{aligned} & \biggl\vert \frac{f(a)+f (a+e^{i \varphi } \eta (b,a) )}{2}-\frac{ \varGamma (\alpha +1)}{2 (e^{i \varphi }\eta (b,a) )^{\alpha }} \bigl[J_{a^{+}}^{\alpha }f \bigl(a+e^{i \varphi }\eta (b,a) \bigr) +J_{ (a+e^{i \varphi }\eta (b,a) )^{-}}^{\alpha }f(a) \bigr] \biggr\vert \\ &\quad \leq \frac{e^{i \varphi }\eta (b,a)}{2} \biggl(\frac{2-2^{1-\alpha p}}{\alpha p+1} \biggr)^{\frac{1}{p}} \biggl[\frac{\pi }{4} \biggl( \bigl\vert f'(a) \bigr\vert ^{\frac{p-1}{p}} +\frac{1-\lambda }{\lambda } \bigl\vert f'(b) \bigr\vert ^{ \frac{p-1}{p}} \biggr) \biggr]^{\frac{p-1}{p}}. \end{aligned}$$

Thus, the proof is done. □

### Remark 7

In Corollary 5, if we choose

- $\eta (b,a)=b-a$ and $\varphi =0$, we get
  $$\begin{aligned} & \biggl\vert \frac{f(a)+f(b)}{2}-\frac{\varGamma (\alpha +1))}{2(b-a)^{\alpha }} \bigl[J_{a^{+}}^{\alpha }f(b)+J_{b^{-}}^{\alpha }f(a) \bigr] \biggr\vert \\&\quad\leq \frac{b-a}{2} \biggl(\frac{2-2^{1-\alpha p}}{\alpha p+1} \biggr) ^{\frac{1}{p}} \biggl[\frac{\pi }{4} \biggl( \bigl\vert f'(a) \bigr\vert ^{ \frac{p-1}{p}} +\frac{1-\lambda }{\lambda } \bigl\vert f'(b) \bigr\vert ^{ \frac{p-1}{p}} \biggr) \biggr]^{\frac{p-1}{p}}. \end{aligned}$$
- $\eta (b,a)=b-a$, $\varphi =0$, and $\lambda =\frac{1}{2}$, we get
  $$\begin{aligned} & \biggl\vert \frac{f(a)+f(b)}{2}-\frac{\varGamma (\alpha +1))}{2(b-a)^{\alpha }} \bigl[J_{a^{+}}^{\alpha }f(b)+J_{b^{-}}^{\alpha }f(a) \bigr] \biggr\vert \\&\quad\leq \frac{b-a}{2} \biggl(\frac{2-2^{1-\alpha p}}{\alpha p+1} \biggr) ^{\frac{1}{p}} \biggl[\frac{\pi }{4} \bigl( \bigl\vert f'(a) \bigr\vert ^{ \frac{p-1}{p}} + \bigl\vert f'(b) \bigr\vert ^{\frac{p-1}{p}} \bigr) \biggr] ^{\frac{p-1}{p}}. \end{aligned}$$

### Corollary 6

*Under the assumptions of Theorem *10. *If* $|f'|^{\frac{p}{p-1}}$ *is* *h*-*convex*, *we have*

$$\begin{aligned} & \biggl\vert \frac{f(a)+f (a+e^{i \varphi } \eta (b,a) )}{2}-\frac{ \varGamma (\alpha +1))}{2 (e^{i \varphi }\eta (b,a) )^{\alpha }} \bigl[J_{a^{+}}^{\alpha }f \bigl(a+e^{i \varphi }\eta (b,a) \bigr) +J_{ (a+e^{i \varphi }\eta (b,a) )^{-}}^{\alpha }f(a) \bigr] \biggr\vert \\ &\quad \leq \frac{e^{i \varphi }\eta (b,a)}{2} \biggl(\frac{2-2^{1-\alpha p}}{\alpha p+1} \biggr)^{\frac{1}{p}} \biggl( \int _{0}^{1} h(t) \biggr) ^{\frac{p-1}{p}} \biggl( \bigl\vert f'(a) \bigr\vert ^{\frac{p-1}{p}} +\frac{1- \lambda }{\lambda } \bigl\vert f'(b) \bigr\vert ^{\frac{p-1}{p}} \biggr). \end{aligned}$$

### Proof

From (9) with $w(t)=1$, we have

$$\begin{aligned} &T_{F,1}(u_{1},u_{2},u_{3}) \\ &\quad =u_{1}- \biggl( \int _{0}^{1} h(t)\,dt \biggr)u_{3} - \frac{1-\lambda }{ \lambda } \biggl( \int _{0}^{1} h(1-t)\,dt \biggr)u_{2} \\ &\quad =u_{1}- \biggl( \int _{0}^{1} h(t)\,dt \biggr)u_{3} - \frac{1-\lambda }{ \lambda } \biggl( \int _{0}^{1} h(t)\,dt \biggr)u_{2} \\ &\quad =u_{1}- \biggl( \int _{0}^{1} h(t)\,dt \biggr) \biggl(u_{2}+ \frac{1- \lambda }{\lambda }u_{3} \biggr) \end{aligned}$$

for $u_{1}, u_{2}, u_{3}\in \mathbb{R}$. So, by Theorem 9, we have

$$\begin{aligned} 0 &\geq T_{F,1} \bigl(G_{1}(f,p), \bigl\vert f'(a) \bigr\vert ^{\frac{p-1}{p}}, \bigl\vert f'(b) \bigr\vert ^{\frac{p-1}{p}} \bigr) \\ &= \biggl(\frac{2}{e^{i \varphi }\eta (b,a)} \biggr)^{\frac{p}{p-1}} \biggl(\frac{\alpha p+1}{2-2^{1-\alpha p}} \biggr)^{\frac{1}{p-1}} \\ &\quad {}\times \biggl\vert \frac{f(a)+f (a+e^{i \varphi } \eta (b,a) )}{2}-\frac{ \varGamma (\alpha +1)}{2 (e^{i \varphi }\eta (b,a) )^{\alpha }} \bigl[J_{a^{+}}^{\alpha }f \bigl(a+e^{i \varphi }\eta (b,a) \bigr) +J_{ (a+e^{i \varphi }\eta (b,a) )^{-}}^{\alpha }f(a) \bigr] \biggr\vert \\ &\quad {}- \biggl( \int _{0}^{1} h(t)\,dt \biggr) \biggl( \bigl\vert f'(a) \bigr\vert ^{ \frac{p-1}{p}} +\frac{1-\lambda }{\lambda } \bigl\vert f'(b) \bigr\vert ^{ \frac{p-1}{p}} \biggr), \end{aligned}$$

that is,

$$\begin{aligned} & \biggl\vert \frac{f(a)+f (a+e^{i \varphi } \eta (b,a) )}{2}-\frac{ \varGamma (\alpha +1)}{2 (e^{i \varphi }\eta (b,a) )^{\alpha }} \bigl[J_{a^{+}}^{\alpha }f \bigl(a+e^{i \varphi }\eta (b,a) \bigr) +J_{ (a+e^{i \varphi }\eta (b,a) )^{-}}^{\alpha }f(a) \bigr] \biggr\vert \\ &\quad \leq \frac{e^{i \varphi }\eta (b,a)}{2} \biggl(\frac{2-2^{1-\alpha p}}{ \alpha p+1} \biggr)^{\frac{1}{p}}\\&\qquad{}\times \biggl( \int _{0}^{1} h(t) \bigl\vert (1-t)^{ \alpha }-t^{\alpha } \bigr\vert \,dt \biggr) \biggl( \bigl\vert f'(a) \bigr\vert ^{\frac{p-1}{p}} +\frac{1-\lambda }{\lambda } \bigl\vert f'(b) \bigr\vert ^{ \frac{p-1}{p}} \biggr). \end{aligned}$$

This completes the proof. □

### Theorem 11

*Let* $I\subseteq \mathbb{R}$ *be an open invex set with respect to bifunction* $\eta : I\times I\to \mathbb{R}$, *where* $\eta (b,a)>0$. *Let* $f: [0,b]\to \mathbb{R}$ *be a differentiable mapping*. *Suppose that* $|f'|^{{\frac{p}{p-1}}}$ *is measurable*, *decreasing*, $\lambda _{\varphi }$-*preinvex function on* *I*, *and* *F*-*convex on* $[a,b]$, *for some* $F\in \mathcal{F}$ *and* $|f'|\in L^{{\frac{p}{p-1}}}(a,b)$. *Then*

15 $$\begin{aligned} &T_{F,w} \bigl(G_{2}(f,p), \bigl\vert f'(a) \bigr\vert ^{\frac{p}{p-1}}, \bigl\vert f'(b) \bigr\vert ^{\frac{p}{p-1}} \bigr)+ \int _{0}^{1} L_{w(t)}\,dt\leq 0, \end{aligned}$$

*where*

$$\begin{aligned} G_{2}(f,p) &= \biggl(\frac{2}{e^{i \varphi }\eta (b,a)} \biggr)^{ \frac{p}{p-1}} \biggl( \frac{\alpha +1}{2-2^{1-\alpha }} \biggr)^{ \frac{1}{p-1}} \biggl\vert \frac{f(a)+f (a+e^{i \varphi } \eta (b,a) )}{2} \\ &\quad {}-\frac{ \varGamma (\alpha +1)}{2 (e^{i \varphi }\eta (b,a) )^{\alpha }} \bigl[J_{a^{+}}^{\alpha }f \bigl(a+e^{i \varphi }\eta (b,a) \bigr) +J_{ (a+e^{i \varphi }\eta (b,a) )^{-}}^{\alpha }f(a) \bigr] \biggr\vert ^{\frac{p}{p-1}} \end{aligned}$$

*for* $w(t)= \vert (1-t)^{\alpha }-t^{\alpha } \vert $.

### Proof

Since $|f'|^{\frac{p}{p-1}}$ is *F*-convex, we have

$$\begin{aligned} F \bigl( \bigl\vert f' \bigl(a+(1-t)e^{i \varphi }\eta (b,a) \bigr) \bigr\vert ^{\frac{p}{p-1}}, \bigl\vert f'(a) \bigr\vert ^{\frac{p}{p-1}}, \bigl\vert f'(b) \bigr\vert ^{\frac{p}{p-1}},t \bigr) \leq 0,\quad t\in [0,1]. \end{aligned}$$

Using (A3) with $w(t)= \vert (1-t)^{\alpha }-t^{\alpha } \vert $, we obtain

$$\begin{aligned} &F \bigl(w(t) \bigl\vert f' \bigl(a+(1-t)e^{i \varphi }\eta (b,a) \bigr) \bigr\vert ^{\frac{p}{p-1}}, w(t) \bigl\vert f'(a) \bigr\vert ^{\frac{p}{p-1}},w(t) \bigl\vert f'(b) \bigr\vert ^{\frac{p}{p-1}},t \bigr) +L_{w(t)}\leq 0,\\ &\quad t\in [0,1]. \end{aligned}$$

Integrating over $[0,1]$ and using axiom (A2), we obtain

$$\begin{aligned} &T_{F,w} \biggl( \int _{0}^{1} w(t) \bigl\vert f' \bigl(a+(1-t)e^{i \varphi } \eta (b,a) \bigr) \bigr\vert ^{\frac{p}{p-1}}\,dt, \bigl\vert f'(a) \bigr\vert ^{\frac{p}{p-1}}, \bigl\vert f'(b) \bigr\vert ^{\frac{p}{p-1}} \biggr)+ \int _{0}^{1} L_{w(t)}\,dt\leq 0,\\ &\quad t\in [0,1]. \end{aligned}$$

Using Lemma 1 and the power mean inequality, we get

$$\begin{aligned} & \biggl\vert \frac{f(a)+f (a+e^{i \varphi } \eta (b,a) )}{2}-\frac{ \varGamma (\alpha +1)}{2 (e^{i \varphi }\eta (b,a) )^{\alpha }} \bigl[J_{a^{+}}^{\alpha }f \bigl(a+e^{i \varphi }\eta (b,a) \bigr) +J_{ (a+e^{i \varphi }\eta (b,a) )^{-}}^{\alpha }f(a) \bigr] \biggr\vert \\ &\quad =\frac{e^{i \varphi }\eta (b,a)}{2} \int _{0}^{1} \bigl\vert (1-t)^{ \alpha }-t^{\alpha } \bigr\vert \bigl\vert f' \bigl(a+(1-t)e^{i \varphi } \eta (b,a) \bigr) \bigr\vert \,dt \\ &\quad \leq \frac{e^{i \varphi }\eta (b,a)}{2} \biggl( \int _{0}^{1} \bigl\vert (1-t)^{ \alpha }-t^{\alpha } \bigr\vert \,dt \biggr)^{\frac{1}{p}} \biggl( \int _{0} ^{1} w(t) \bigl\vert f' \bigl(a+(1-t)e^{i \varphi }\eta (b,a) \bigr) \bigr\vert ^{\frac{p}{p-1}}\,dt \biggr)^{\frac{p-1}{p}} \\ &\quad = \frac{e^{i \varphi }\eta (b,a)}{2} \biggl(\frac{2-2^{1-\alpha }}{ \alpha +1} \biggr)^{\frac{1}{p}} \biggl( \int _{0}^{1} w(t) \bigl\vert f' \bigl(a+(1-t)e^{i \varphi }\eta (b,a) \bigr) \bigr\vert ^{\frac{p}{p-1}}\,dt \biggr) ^{ \frac{p-1}{p}} \end{aligned}$$

or, equivalently,

$$\begin{aligned} & \biggl(\frac{2}{e^{i \varphi }\eta (b,a)} \biggr)^{\frac{p}{p-1}} \biggl(\frac{\alpha +1}{2-2^{1-\alpha }} \biggr)^{\frac{1}{p-1}} \biggl\vert \frac{f(a)+f (a+e^{i \varphi } \eta (b,a) )}{2} \\ &\qquad {}-\frac{ \varGamma (\alpha +1)}{2 (e^{i \varphi }\eta (b,a) )^{\alpha }} \bigl[J_{a^{+}}^{\alpha }f \bigl(a+e^{i \varphi }\eta (b,a) \bigr) +J_{ (a+e^{i \varphi }\eta (b,a) )^{-}}^{\alpha }f(a) \bigr] \biggr\vert ^{\frac{p}{p-1}} \\ &\quad \leq \int _{0}^{1} w(t) \bigl\vert f' \bigl(a+(1-t)e^{i \varphi }\eta (b,a) \bigr) \bigr\vert ^{\frac{p}{p-1}}\,dt. \end{aligned}$$

Because $T_{F,w}$ is nondecreasing with respect to the first variable, we find

$$\begin{aligned} &T_{F,w} \bigl(G_{2}(f,p), \bigl\vert f'(a) \bigr\vert ^{{\frac{p}{p-1}}}, \bigl\vert f'(b) \bigr\vert ^{{\frac{p}{p-1}}} \bigr)+ \int _{0}^{1} L_{w(t)}\,dt \leq 0. \end{aligned}$$

This completes the proof. □

### Remark 8

If we choose $\eta (b,a)=b-a$ and $\varphi =0$ in Theorem 11, we get

$$\begin{aligned} &T_{F,w} \biggl( \biggl(\frac{2}{b-a} \biggr)^{\frac{p}{p-1}} \biggl(\frac{ \alpha +1}{2-2^{1-\alpha }} \biggr)^{{\frac{1}{p-1}}} \biggl\vert \frac{f(a)+f(b)}{2} - \frac{\varGamma (\alpha +1))}{2(b-a)^{\alpha }} \bigl[J_{a^{+}}^{\alpha }f(b)+J_{b^{-}}^{\alpha }f(a) \bigr] \biggr\vert , \\ &\quad \bigl\vert f'(a) \bigr\vert ,\bigl\vert f'(b) \bigr\vert \biggr)+ \int _{0}^{1} L_{w(t)}\,dt\leq 0. \end{aligned}$$

### Corollary 7

*Under the assumptions of Theorem *11, *if* $|f'|^{\frac{p}{p-1}}$ *is* *ε*-*convex*, *we have*

$$\begin{aligned} & \biggl\vert \frac{f(a)+f (a+e^{i \varphi } \eta (b,a) )}{2}-\frac{ \varGamma (\alpha +1))}{2 (e^{i \varphi }\eta (b,a) )^{\alpha }} \bigl[J_{a^{+}}^{\alpha }f \bigl(a+e^{i \varphi }\eta (b,a) \bigr) +J_{ (a+e^{i \varphi }\eta (b,a) )^{-}}^{\alpha }f(a) \bigr] \biggr\vert \\ &\quad \leq \frac{e^{i \varphi }\eta (b,a)}{2} \biggl(\frac{2-2^{1-\alpha }}{ \alpha +1} \biggr)^{\frac{1}{p}} \biggl[\frac{2^{\alpha }-1}{2^{ \alpha }(\alpha +1)} \bigl( \bigl\vert f'(a) \bigr\vert ^{\frac{p}{p-1}} + \bigl\vert f'(b) \bigr\vert ^{\frac{p}{p-1}} \bigr) +2\varepsilon \biggr] ^{\frac{p-1}{p}}. \end{aligned}$$

### Proof

Using (5) with $w(t)= \vert (1-t)^{\alpha }-t^{\alpha } \vert $, we get

$$\begin{aligned} \int _{0}^{1} L_{w(t)}\,dt &=\varepsilon \biggl(1-2\frac{2^{\alpha }-1}{2^{ \alpha }(\alpha +1)} \biggr). \end{aligned}$$

From (4) with $w(t)= \vert (1-t)^{\alpha }-t^{\alpha } \vert $, we get

$$\begin{aligned} &T_{F,w}(u_{1},u_{2},u_{3}) \\ &\quad =u_{1}- \biggl( \int _{0}^{1} \bigl\vert (1-t)^{\alpha }-t^{\alpha } \bigr\vert t\,dt \biggr)u _{2} - \biggl( \int _{0}^{1} \bigl\vert (1-t)^{\alpha }-t^{\alpha } \bigr\vert (1-t)\,dt \biggr)u _{3}-\varepsilon \\ &\quad =u_{1}-\frac{2^{\alpha }-1}{2^{\alpha }(\alpha +1)}(u_{2}+u_{3})- \varepsilon , \end{aligned}$$

for $u_{1}, u_{2}, u_{3}\in \mathbb{R}$. Hence, by Theorem 10, we have

$$\begin{aligned} 0 &\geq T_{F,w} \bigl(G_{2}(f,p), \bigl\vert f'(a) \bigr\vert ^{\frac{p}{p-1}}, \bigl\vert f'(b) \bigr\vert ^{\frac{p}{p-1}} \bigr)+ \int _{0}^{1} L_{w(t)}\,dt \\ &=G_{2}(f,p)-\frac{2^{\alpha }-1}{2^{\alpha }(\alpha +1)} \bigl( \bigl\vert f'(a) \bigr\vert ^{\frac{p}{p-1}}+ \bigl\vert f'(b) \bigr\vert ^{\frac{p}{p-1}} \bigr) -\varepsilon +\varepsilon \biggl(1-2\frac{2^{\alpha }-1}{2^{\alpha }(\alpha +1)} \biggr). \end{aligned}$$

This implies that

$$\begin{aligned} & \biggl\vert \frac{f(a)+f (a+e^{i \varphi } \eta (b,a) )}{2}-\frac{ \varGamma (\alpha +1))}{2 (e^{i \varphi }\eta (b,a) )^{\alpha }} \bigl[J_{a^{+}}^{\alpha }f \bigl(a+e^{i \varphi }\eta (b,a) \bigr) +J_{ (a+e^{i \varphi }\eta (b,a) )^{-}}^{\alpha }f(a) \bigr] \biggr\vert \\ &\quad \leq \frac{e^{i \varphi }\eta (b,a)}{2} \biggl(\frac{2-2^{1-\alpha }}{\alpha +1} \biggr)^{\frac{1}{p}} \biggl[\frac{2^{\alpha }-1}{2^{ \alpha }(\alpha +1)} \bigl( \bigl\vert f'(a) \bigr\vert ^{\frac{p}{p-1}} + \bigl\vert f'(b) \bigr\vert ^{\frac{p}{p-1}} \bigr)+2\varepsilon \biggr] ^{\frac{p-1}{p}}. \end{aligned}$$

This completes the proof. □

### Remark 9

In Corollary 7, if we choose

- $\eta (b,a)=b-a$ and $\varphi =0$, we get
  $$\begin{aligned} & \biggl\vert \frac{f(a)+f(b)}{2}-\frac{\varGamma (\alpha +1))}{2(b-a)^{\alpha }} \bigl[J_{a^{+}}^{\alpha }f(b)+J_{b^{-}}^{\alpha }f(a) \bigr] \biggr\vert \\ &\quad \leq \frac{b-a}{2} \biggl(\frac{2-2^{1-\alpha }}{\alpha +1} \biggr) ^{\frac{1}{p}} \biggl[\frac{2^{\alpha }-1}{2^{\alpha }(\alpha +1)} \bigl( \bigl\vert f'(a) \bigr\vert ^{\frac{p}{p-1}} + \bigl\vert f'(b) \bigr\vert ^{ \frac{p}{p-1}} \bigr)+2\varepsilon \biggr]^{\frac{p-1}{p}}. \end{aligned}$$
- $\eta (b,a)=b-a$, $\varphi =0$, and $\varepsilon =0$, we get
  $$\begin{aligned} & \biggl\vert \frac{f(a)+f(b)}{2}-\frac{\varGamma (\alpha +1))}{2(b-a)^{\alpha }} \bigl[J_{a^{+}}^{\alpha }f(b)+J_{b^{-}}^{\alpha }f(a) \bigr] \biggr\vert \\ &\quad \leq \frac{b-a}{2} \biggl(\frac{2-2^{1-\alpha }}{\alpha +1} \biggr) ^{\frac{1}{p}} \biggl[\frac{2^{\alpha }-1}{2^{\alpha }(\alpha +1)} \bigl( \bigl\vert f'(a) \bigr\vert ^{\frac{p}{p-1}} + \bigl\vert f'(b) \bigr\vert ^{ \frac{p}{p-1}} \bigr) \biggr]^{\frac{p-1}{p}}. \end{aligned}$$

### Corollary 8

*Under the assumptions of Theorem *11. *If* $|f'|^{\frac{p-1}{p}}$ *is* $\lambda _{\varphi }$-*preinvex*, *we have*

$$\begin{aligned} & \biggl\vert \frac{f(a)+f (a+e^{i \varphi } \eta (b,a) )}{2}-\frac{ \varGamma (\alpha +1))}{2 (e^{i \varphi }\eta (b,a) )^{\alpha }} \bigl[J_{a^{+}}^{\alpha }f \bigl(a+e^{i \varphi }\eta (b,a) \bigr) +J_{ (a+e^{i \varphi }\eta (b,a) )^{-}}^{\alpha }f(a) \bigr] \biggr\vert \\ &\quad \leq \frac{e^{i \varphi }\eta (b,a)}{2} \biggl(\frac{2-2^{1-\alpha }}{ \alpha +1} \biggr)^{\frac{1}{p}}\\&\qquad{}\times \biggl[\frac{B_{\frac{1}{2}} (\frac{1}{2}, \alpha +\frac{1}{2} ) -B_{\frac{1}{2}} (\alpha + \frac{1}{2},\frac{1}{2} )}{2} \biggl( \bigl\vert f'(a) \bigr\vert ^{ \frac{p}{p-1}}+\frac{1-\lambda }{\lambda } \bigl\vert f'(b) \bigr\vert ^{ \frac{p}{p-1}} \biggr) \biggr]^{\frac{p-1}{p}}. \end{aligned}$$

### Proof

Using (7) with $w(t)= \vert (1-t)^{\alpha }-t^{\alpha } \vert $, we have

$$\begin{aligned} &T_{F,w}(u_{1},u_{2},u_{3}) \\ &\quad =u_{1}- \biggl( \int _{0}^{1} \frac{\sqrt{t}}{2\sqrt{1-t}} \bigl\vert (1-t)^{ \alpha }-t^{\alpha } \bigr\vert \,dt \biggr)u_{3}- \frac{1-\lambda }{\lambda } \biggl( \int _{0}^{1} \frac{\sqrt{1-t}}{2\sqrt{t}} \bigl\vert (1-t)^{ \alpha }-t^{\alpha } \bigr\vert \,dt \biggr)u_{2} \\ &\quad =u_{1}-\frac{1}{2} \biggl(B_{\frac{1}{2}} \biggl( \frac{1}{2},\alpha + \frac{1}{2} \biggr) -B_{\frac{1}{2}} \biggl( \alpha +\frac{1}{2}, \frac{1}{2} \biggr) \biggr) \biggl(u_{2}+ \frac{1-\lambda }{\lambda }u _{3} \biggr) \end{aligned}$$

for $u_{1}, u_{2}, u_{3}\in \mathbb{R}$. Now, by Theorem 11, we have

$$\begin{aligned} 0 &\geq T_{F,w} \bigl(G_{2}(f,p), \bigl\vert f'(a) \bigr\vert ^{\frac{p-1}{p}}, \bigl\vert f'(b) \bigr\vert ^{\frac{p-1}{p}} \bigr) \\ &= \biggl(\frac{2}{e^{i \varphi }\eta (b,a)} \biggr)^{\frac{p}{p-1}} \biggl(\frac{\alpha +1}{2-2^{1-\alpha }} \biggr)^{\frac{1}{p-1}} \\ &\quad {}\times \biggl\vert \frac{f(a)+f (a+e^{i \varphi } \eta (b,a) )}{2}-\frac{ \varGamma (\alpha +1)}{2 (e^{i \varphi }\eta (b,a) )^{\alpha }} \bigl[J_{a^{+}}^{\alpha }f \bigl(a+e^{i \varphi }\eta (b,a) \bigr) +J_{ (a+e^{i \varphi }\eta (b,a) )^{-}}^{\alpha }f(a) \bigr] \biggr\vert \\ &\quad {}-\frac{1}{2} \biggl(B_{\frac{1}{2}} \biggl(\frac{1}{2}, \alpha + \frac{1}{2} \biggr) -B_{\frac{1}{2}} \biggl(\alpha + \frac{1}{2}, \frac{1}{2} \biggr) \biggr) \biggl( \bigl\vert f'(a) \bigr\vert ^{ \frac{p-1}{p}} +\frac{1-\lambda }{\lambda } \bigl\vert f'(b) \bigr\vert ^{ \frac{p-1}{p}} \biggr). \end{aligned}$$

This leads to

$$\begin{aligned} & \biggl\vert \frac{f(a)+f (a+e^{i \varphi } \eta (b,a) )}{2}-\frac{ \varGamma (\alpha +1)}{2 (e^{i \varphi }\eta (b,a) )^{\alpha }} \bigl[J_{a^{+}}^{\alpha }f \bigl(a+e^{i \varphi }\eta (b,a) \bigr) +J_{ (a+e^{i \varphi }\eta (b,a) )^{-}}^{\alpha }f(a) \bigr] \biggr\vert \\ &\quad \leq \frac{e^{i \varphi }\eta (b,a)}{2} \biggl(\frac{2-2^{1-\alpha }}{ \alpha +1} \biggr)^{\frac{1}{p}}\\&\qquad{}\times \biggl[\frac{B_{\frac{1}{2}} (\frac{1}{2}, \alpha +\frac{1}{2} ) -B_{\frac{1}{2}} (\alpha + \frac{1}{2},\frac{1}{2} )}{2} \biggl( \bigl\vert f'(a) \bigr\vert ^{ \frac{p}{p-1}}+\frac{1-\lambda }{\lambda } \bigl\vert f'(b) \bigr\vert ^{ \frac{p}{p-1}} \biggr) \biggr]^{\frac{p-1}{p}}. \end{aligned}$$

Thus, the proof is done. □

### Remark 10

In Corollary 8, if we choose

- $\eta (b,a)=b-a$ and $\varphi =0$, we get
  $$\begin{aligned} & \biggl\vert \frac{f(a)+f(b)}{2}-\frac{\varGamma (\alpha +1))}{2(b-a)^{\alpha }} \bigl[J_{a^{+}}^{\alpha }f(b)+J_{b^{-}}^{\alpha }f(a) \bigr] \biggr\vert \\ &\quad \leq \frac{b-a}{2} \biggl(\frac{2-2^{1-\alpha }}{\alpha +1} \biggr) ^{\frac{1}{p}}\\&\qquad{}\times \biggl[\frac{B_{\frac{1}{2}} (\frac{1}{2},\alpha +\frac{1}{2} ) -B_{\frac{1}{2}} (\alpha +\frac{1}{2}, \frac{1}{2} )}{2} \biggl( \bigl\vert f'(a) \bigr\vert ^{\frac{p}{p-1}}+\frac{1- \lambda }{\lambda } \bigl\vert f'(b) \bigr\vert ^{\frac{p}{p-1}} \biggr) \biggr] ^{\frac{p-1}{p}}. \end{aligned}$$
- $\eta (b,a)=b-a$, $\varphi =0$, and $\lambda =\frac{1}{2}$, we get
  $$\begin{aligned} & \biggl\vert \frac{f(a)+f(b)}{2}-\frac{\varGamma (\alpha +1))}{2(b-a)^{\alpha }} \bigl[J_{a^{+}}^{\alpha }f(b)+J_{b^{-}}^{\alpha }f(a) \bigr] \biggr\vert \\ &\quad \leq \frac{b-a}{2} \biggl(\frac{2-2^{1-\alpha }}{\alpha +1} \biggr) ^{\frac{1}{p}}\\&\qquad{}\times \biggl[\frac{B_{\frac{1}{2}} (\frac{1}{2},\alpha +\frac{1}{2} ) -B_{\frac{1}{2}} (\alpha +\frac{1}{2}, \frac{1}{2} )}{2} \bigl( \bigl\vert f'(a) \bigr\vert ^{\frac{p}{p-1}}+ \bigl\vert f'(b) \bigr\vert ^{\frac{p}{p-1}} \bigr) \biggr]^{\frac{p-1}{p}}. \end{aligned}$$

### Corollary 9

*Under the assumptions of Theorem *11. *If* $|f'|^{\frac{p}{p-1}}$ *is* *h*-*convex*, *we have*

$$\begin{aligned} & \biggl\vert \frac{f(a)+f (a+e^{i \varphi } \eta (b,a) )}{2}-\frac{ \varGamma (\alpha +1))}{2 (e^{i \varphi }\eta (b,a) )^{\alpha }} \bigl[J_{a^{+}}^{\alpha }f \bigl(a+e^{i \varphi }\eta (b,a) \bigr) +J_{ (a+e^{i \varphi }\eta (b,a) )^{-}}^{\alpha }f(a) \bigr] \biggr\vert \\ &\quad \leq \frac{e^{i \varphi }\eta (b,a)}{2} \biggl(\frac{2-2^{1-\alpha }}{\alpha +1} \biggr)^{\frac{1}{p}}\\&\qquad{}\times \biggl( \int _{0}^{1} h(t) \bigl\vert (1-t)^{ \alpha }-t^{\alpha } \bigr\vert \,dt \biggr)^{\frac{p-1}{p}} \biggl( \bigl\vert f'(a) \bigr\vert ^{\frac{p}{p-1}}+\frac{1-\lambda }{\lambda } \bigl\vert f'(b) \bigr\vert ^{ \frac{p}{p-1}} \biggr)^{\frac{p-1}{p}}. \end{aligned}$$

### Proof

From (9) with $w(t)= \vert (1-t)^{\alpha }-t^{\alpha } \vert $, we have

$$\begin{aligned} &T_{F,w}(u_{1},u_{2},u_{3}) \\ &\quad =u_{1}- \biggl( \int _{0}^{1} h(t) \bigl\vert (1-t)^{\alpha }-t^{\alpha } \bigr\vert \,dt \biggr)u _{3} -\frac{1-\lambda }{\lambda } \biggl( \int _{0}^{1} h(1-t) \bigl\vert (1-t)^{ \alpha }-t^{\alpha } \bigr\vert \,dt \biggr)u_{2} \\ &\quad =u_{1}- \biggl( \int _{0}^{1} h(t) \bigl\vert (1-t)^{\alpha }-t^{\alpha } \bigr\vert \,dt \biggr)u _{3} -\frac{1-\lambda }{\lambda } \biggl( \int _{0}^{1} h(t) \bigl\vert (1-t)^{ \alpha }-t^{\alpha } \bigr\vert \,dt \biggr)u_{2} \\ &\quad =u_{1}- \biggl( \int _{0}^{1} h(t) \bigl\vert (1-t)^{\alpha }-t^{\alpha } \bigr\vert \,dt \biggr) \biggl(u_{2}+\frac{1- \lambda }{\lambda }u_{3} \biggr) \end{aligned}$$

for $u_{1}, u_{2}, u_{3}\in \mathbb{R}$. So, by Theorem 11, we have

$$\begin{aligned} 0 &\geq T_{F,w} \bigl(G_{2}(f,p), \bigl\vert f'(a) \bigr\vert ^{\frac{p-1}{p}}, \bigl\vert f'(b) \bigr\vert ^{\frac{p-1}{p}} \bigr) \\ &= \biggl(\frac{2}{e^{i \varphi }\eta (b,a)} \biggr)^{\frac{p}{p-1}} \biggl(\frac{\alpha +1}{2-2^{1-\alpha }} \biggr)^{\frac{1}{p-1}} \\ &\quad {}\times \biggl\vert \frac{f(a)+f (a+e^{i \varphi } \eta (b,a) )}{2}-\frac{ \varGamma (\alpha +1)}{2 (e^{i \varphi }\eta (b,a) )^{\alpha }} \bigl[J_{a^{+}}^{\alpha }f \bigl(a+e^{i \varphi }\eta (b,a) \bigr) +J_{ (a+e^{i \varphi }\eta (b,a) )^{-}}^{\alpha }f(a) \bigr] \biggr\vert \\ &\quad {}- \biggl( \int _{0}^{1} h(t)\,dt \biggr) \biggl( \bigl\vert f'(a) \bigr\vert ^{ \frac{p-1}{p}} +\frac{1-\lambda }{\lambda } \bigl\vert f'(b) \bigr\vert ^{ \frac{p-1}{p}} \biggr), \end{aligned}$$

that is,

$$\begin{aligned} & \biggl\vert \frac{f(a)+f (a+e^{i \varphi } \eta (b,a) )}{2}-\frac{ \varGamma (\alpha +1)}{2 (e^{i \varphi }\eta (b,a) )^{\alpha }} \bigl[J_{a^{+}}^{\alpha }f \bigl(a+e^{i \varphi }\eta (b,a) \bigr) +J_{ (a+e^{i \varphi }\eta (b,a) )^{-}}^{\alpha }f(a) \bigr] \biggr\vert \\ &\quad \leq \frac{e^{i \varphi }\eta (b,a)}{2} \biggl(\frac{2-2^{1-\alpha }}{\alpha +1} \biggr)^{\frac{1}{p}}\\&\qquad{}\times \biggl( \int _{0}^{1} h(t) \bigl\vert (1-t)^{ \alpha }-t^{\alpha } \bigr\vert \,dt \biggr)^{\frac{p-1}{p}} \biggl( \bigl\vert f'(a) \bigr\vert ^{\frac{p}{p-1}}+\frac{1-\lambda }{\lambda } \bigl\vert f'(b) \bigr\vert ^{ \frac{p}{p-1}} \biggr)^{\frac{p-1}{p}}. \end{aligned}$$

This completes the proof. □

## Trapezoid type inequalities for twice differentiable functions

In this section, we establish some trapezoid type inequalities for functions whose second derivatives absolutely values are

### Theorem 12

*Let* $f: [0,b]\to \mathbb{R}$ *be a differentiable mapping and* $|f''|$ *is measurable*, *decreasing*, $\lambda _{\varphi }$-*preinvex function on* $[0,b]$ *for* $0\leq a< b$, $\eta (b,a)>0$ *and* $\alpha >0$. *Suppose that* *F*-*convex on* $[0,b]$, *for some* $F\in \mathcal{F}$ *and the function* $t\in (0,1)\to L_{w(t)}$ *belongs to* $L^{1}(0,1)$, *where* $w(t)=1-(1-t)^{\alpha +1}-t^{\alpha +1}$. *Then*

16 $$\begin{aligned} \begin{aligned}[b] &T_{F,w} \biggl(\frac{2(\alpha +1)}{ (e^{i \varphi }\eta (b,a) ) ^{2}} \biggl\vert \frac{f(a)+f (a+e^{i \varphi }\eta (b,a) )}{2} \\&\quad{}-\frac{\varGamma (\alpha +1)}{2 (e^{i \varphi }\eta (b,a) ) ^{\alpha }} \bigl[J_{a^{+}}^{\alpha }f \bigl(a+e^{i \varphi }\eta (b,a) \bigr) +J_{ (a+e^{i \varphi }\eta (b,a) ) ^{-}}^{\alpha }f(a) \bigr] \biggr\vert ,\bigl\vert f''(a) \bigr\vert , \bigl\vert f''(b) \bigr\vert \biggr) \\ &\quad {} + \int _{0}^{1} L _{w(t)}\,dt\leq 0. \end{aligned} \end{aligned}$$

### Proof

Since $|f''|$ is *F*-convex, we can see that

$$\begin{aligned} F \bigl( \bigl\vert f'' \bigl(a+(1-t)e^{i \varphi } \eta (b,a) \bigr) \bigr\vert , \bigl\vert f''(a) \bigr\vert , \bigl\vert f''(b) \bigr\vert ,t \bigr)\leq 0,\quad t \in [0,1]. \end{aligned}$$

Multiplying this inequality by $w(t)=1-(1-t)^{\alpha +1}-t^{\alpha +1}$ and using axiom (A3), we have

$$\begin{aligned} F \bigl(w(t) \bigl\vert f'' \bigl(a+(1-t)e^{i \varphi } \eta (b,a) \bigr) \bigr\vert ,w(t) \bigl\vert f''(a) \bigr\vert ,w(t) \bigl\vert f''(b) \bigr\vert ,t \bigr)+L_{w(t)}\leq 0,\quad t\in [0,1]. \end{aligned}$$

Integrating over $[0,1]$ and using axiom (A2), we get

$$\begin{aligned} &T_{F,w} \biggl( \int _{0}^{1} w(t) \bigl\vert f'' \bigl(a+(1-t)e^{i \varphi } \eta (b,a) \bigr) \bigr\vert \,dt, \bigl\vert f''(a) \bigr\vert , \bigl\vert f''(b) \bigr\vert ,t \biggr) + \int _{0}^{1} L_{w(t)}\,dt\leq 0,\\ &\quad t\in [0,1]. \end{aligned}$$

Using Lemma 2, we have

$$\begin{aligned} &\frac{2(\alpha +1)}{ (e^{i \varphi }\eta (b,a) )^{2}} \biggl\vert \frac{f(a)+f (a+e^{i \varphi }\eta (b,a) )}{2} \\ &\qquad {}-\frac{ \varGamma (\alpha +1))}{2 (e^{i \varphi }\eta (b,a) )^{\alpha }} \bigl[J_{a^{+}}^{\alpha }f \bigl(a+e^{i \varphi }\eta (b,a) \bigr) +J_{ (a+e^{i \varphi }\eta (b,a) )^{-}}^{\alpha }f(a) \bigr] \biggr\vert \\ &\quad \leq \int _{0}^{1} \bigl[1-(1-t)^{\alpha +1}-t^{\alpha +1} \bigr] \bigl\vert f'' \bigl(a+(1-t)e^{i \varphi } \eta (b,a) \bigr) \bigr\vert \,dt. \end{aligned}$$

Because $T_{F,w}$ is nondecreasing with respect to the first variable so that

$$\begin{aligned} &T_{F,w} \biggl(\frac{2(\alpha +1)}{ (e^{i \varphi }\eta (b,a) ) ^{2}} \biggl\vert \frac{f(a)+ f (a+e^{i \varphi }\eta (b,a) )}{2}\\ &\quad {}- \frac{ \varGamma (\alpha +1)}{2 (e^{i \varphi } \eta (b,a) )^{\alpha }} \bigl[J_{a^{+}}^{\alpha }f \bigl(a+e^{i \varphi } \eta (b,a) \bigr) +J_{ (a+e^{i \varphi }\eta (b,a) )^{-}}^{\alpha }f(a) \bigr] \biggr\vert ,\bigl\vert f''(a) \bigr\vert , \bigl\vert f''(b) \bigr\vert \biggr) \\ &\quad {} + \int _{0}^{1} L _{w(t)}\,dt\leq 0,\quad t\in [0,1]. \end{aligned}$$

This completes the proof. □

### Remark 11

By taking $\eta (b,a)=b-a$ and $\varphi =0$ in Theorem 12, we obtain

$$\begin{aligned} &T_{F,w} \biggl(\frac{2(\alpha +1)}{(b-a)^{2}} \biggl\vert \frac{f(a)+f(b)}{2} - \frac{\varGamma (\alpha +1))}{2(b-a)^{\alpha }} \bigl[J_{a^{+}}^{\alpha }f(b)+J_{b^{-}}^{\alpha }f(a) \bigr] \biggr\vert , \bigl\vert f''(a) \bigr\vert , \bigl\vert f''(b) \bigr\vert \biggr)\\ &\quad {}+ \int _{0}^{1} L_{w(t)}\,dt\leq 0. \end{aligned}$$

### Corollary 10

*Under the assumptions of Theorem *12, *if* $|f''|$ *is* *ε*-*convex*, *then*

$$\begin{aligned} & \biggl\vert \frac{f(a)+f (a+e^{i \varphi } \eta (b,a) )}{2}-\frac{ \varGamma (\alpha +1))}{2 (e^{i \varphi }\eta (b,a) )^{\alpha }} \bigl[J_{a^{+}}^{\alpha }f \bigl(a+e^{i \varphi }\eta (b,a) \bigr) +J_{ (a+e^{i \varphi }\eta (b,a) )^{-}}^{\alpha }f(a) \bigr] \biggr\vert \\ &\quad \leq \frac{\alpha (e^{i \varphi }\eta (b,a) )^{2}}{4( \alpha +1)(\alpha +2)} \bigl( \bigl\vert f''(a) \bigr\vert + \bigl\vert f''(b) \bigr\vert +2 \varepsilon \bigr). \end{aligned}$$

### Proof

Using (5) with $w(t)=1-(1-t)^{\alpha +1}-t^{\alpha +1}$, we find

17 $$\begin{aligned} \int _{0}^{1} L_{w(t)}\,dt &=\varepsilon \int _{0}^{1} \bigl((1-t)^{ \alpha +1}+t^{\alpha +1} \bigr)\,dt =\frac{2\varepsilon }{\alpha +2}. \end{aligned}$$

With $w(t)=1-(1-t)^{\alpha +1}-t^{\alpha +1}$, Eq. (4) gives

18 $$\begin{aligned} &T_{F,w}(u_{1},u_{2},u_{3}) \\ &\quad =u_{1}- \biggl( \int _{0}^{1} t \bigl[1-(1-t)^{\alpha +1}-t^{\alpha +1} \bigr]\,dt \biggr)u_{2} \\ &\qquad{}- \biggl( \int _{0}^{1} (1-t) \bigl[1-(1-t)^{ \alpha +1}-t^{\alpha +1} \bigr]\,dt \biggr)u_{3}-\varepsilon \\ &\quad =u_{1}-\frac{\alpha }{2(\alpha +2)}(u_{2}+u_{3})- \varepsilon , \end{aligned}$$

for $u_{1}, u_{2}, u_{3}\in \mathbb{R}$. Hence, by Theorem 12, we have

$$\begin{aligned} 0 &\geq T_{F,w} \biggl(\frac{2(\alpha +1)}{ (e^{i \varphi }\eta (b,a) ) ^{2}} \biggl\vert \frac{f(a)+f (a+e^{i \varphi }\eta (b,a) )}{2} \\ &\quad {}-\frac{\varGamma (\alpha +1)}{2 (e^{i \varphi }\eta (b,a) ) ^{\alpha }} \bigl[J_{a^{+}}^{\alpha }f \bigl(a+e^{i \varphi }\eta (b,a) \bigr) +J_{ (a+e^{i \varphi }\eta (b,a) ) ^{-}}^{\alpha }f(a) \bigr] \biggr\vert ,\bigl\vert f''(a) \bigr\vert , \bigl\vert f''(b) \bigr\vert \biggr) \\ &\quad {}+ \int _{0}^{1} L _{w(t)}\,dt \\ &=\frac{2(\alpha +1)}{ (e^{i \varphi }\eta (b,a) )^{2}} \biggl\vert \frac{f(a)+f (a+e^{i \varphi }\eta (b,a) )}{2}\\ &\quad {} -\frac{ \varGamma (\alpha +1)}{2 (e^{i \varphi }\eta (b,a) )^{\alpha }} \bigl[J_{a^{+}}^{\alpha }f \bigl(a+e^{i \varphi }\eta (b,a) \bigr) +J_{ (a+e^{i \varphi }\eta (b,a) )^{-}}^{\alpha }f(a) \bigr] \biggr\vert \\ &\quad{}-\frac{\alpha }{2(\alpha +2)} \bigl( \bigl\vert f''(a) \bigr\vert + \bigl\vert f''(b) \bigr\vert \bigr) - \varepsilon +\frac{2\varepsilon }{\alpha +2} \\ &=\frac{2(\alpha +1)}{ (e^{i \varphi }\eta (b,a) )^{2}} \biggl\vert \frac{f(a)+f (a+e^{i \varphi }\eta (b,a) )}{2}\\ &\quad {} -\frac{ \varGamma (\alpha +1)}{2 (e^{i \varphi }\eta (b,a) )^{\alpha }} \bigl[J_{a^{+}}^{\alpha }f \bigl(a+e^{i \varphi }\eta (b,a) \bigr) +J_{ (a+e^{i \varphi }\eta (b,a) )^{-}}^{\alpha }f(a) \bigr] \biggr\vert \\ &\quad{}-\frac{\alpha }{2(\alpha +2)} \bigl( \bigl\vert f''(a) \bigr\vert + \bigl\vert f''(b) \bigr\vert \bigr) -\frac{ \alpha }{\alpha +2}\varepsilon . \end{aligned}$$

This completes the proof. □

### Remark 12

In Corollary 10, if we take

- $\eta (b,a)=b-a$ and $\varphi =0$, we get
  $$\begin{aligned} & \biggl\vert \frac{f(a)+f(b)}{2}-\frac{\varGamma (\alpha +1))}{2(b-a)^{\alpha }} \bigl[J_{a^{+}}^{\alpha }f(b)+J_{b^{-}}^{\alpha }f(a) \bigr] \biggr\vert \\ &\quad \leq \frac{\alpha (b-a)^{2}}{4(\alpha +1)(\alpha +2)} \bigl( \bigl\vert f''(a) \bigr\vert + \bigl\vert f''(b) \bigr\vert +2\varepsilon \bigr). \end{aligned}$$
- $\eta (b,a)=b-a$, $\varphi =0$, and $\varepsilon =0$, we get
  $$\begin{aligned} & \biggl\vert \frac{f(a)+f(b)}{2}-\frac{\varGamma (\alpha +1))}{2(b-a)^{\alpha }} \bigl[J_{a^{+}}^{\alpha }f(b)+J_{b^{-}}^{\alpha }f(a) \bigr] \biggr\vert \leq \frac{\alpha (b-a)^{2}}{4(\alpha +1)(\alpha +2)} \bigl( \bigl\vert f''(a) \bigr\vert + \bigl\vert f''(b) \bigr\vert \bigr). \end{aligned}$$

### Corollary 11

*Under the assumptions of Theorem *12, *if* $|f''|$ *is* $\lambda _{\varphi }$-*preinvex*, *then*

$$\begin{aligned} & \biggl\vert \frac{f(a)+f (a+e^{i \varphi } \eta (b,a) )}{2}-\frac{ \varGamma (\alpha +1))}{2 (e^{i \varphi }\eta (b,a) )^{\alpha }} \bigl[J_{a^{+}}^{\alpha }f \bigl(a+e^{i \varphi }\eta (b,a) \bigr) +J_{ (a+e^{i \varphi }\eta (b,a) )^{-}}^{\alpha }f(a) \bigr] \biggr\vert \\ &\quad \leq \frac{ (e^{i \varphi }\eta (b,a) )^{2}}{2(\alpha +1)} \biggl[\frac{\pi }{2}-\beta \biggl( \frac{1}{2},\alpha +\frac{5}{2} \biggr) -\beta \biggl( \frac{3}{2},\alpha +\frac{3}{2} \biggr) \biggr] \biggl( \bigl\vert f''(a) \bigr\vert +\frac{1- \lambda }{\lambda } \bigl\vert f''(b) \bigr\vert \biggr). \end{aligned}$$

### Proof

Using (7) with $w(t)=1-(1-t)^{\alpha +1}-t^{\alpha +1}$, we have

19 $$\begin{aligned} \begin{aligned}[b] &T_{F,w}(u_{1},u_{2},u_{3}) \\ &\quad =u_{1}- \biggl( \int _{0}^{1} \frac{\sqrt{t}}{2\sqrt{1-t}} \bigl[1-(1-t)^{ \alpha +1}-t^{\alpha +1} \bigr]\,dt \biggr)u_{3}\\ &\qquad{}- \frac{1-\lambda }{ \lambda } \biggl( \int _{0}^{1} \frac{\sqrt{1-t}}{2\sqrt{t}} \bigl[1-(1-t)^{ \alpha +1}-t^{\alpha +1} \bigr]\,dt \biggr)u_{2} \\ &\quad =u_{1}- \biggl[\frac{\pi }{2}-\beta \biggl(\frac{1}{2}, \alpha + \frac{5}{2} \biggr) -\beta \biggl(\frac{3}{2},\alpha + \frac{3}{2} \biggr) \biggr] \biggl(u _{2}+\frac{1-\lambda }{\lambda }u_{3} \biggr) \end{aligned} \end{aligned}$$

for $u_{1}, u_{2}, u_{3}\in \mathbb{R}$. Hence, by Theorem 12, we get

$$\begin{aligned} 0 &\geq T_{F,w} \biggl(\frac{2(\alpha +1)}{ (e^{i \varphi }\eta (b,a) ) ^{2}} \biggl\vert \frac{f(a)+f (a+e^{i \varphi }\eta (b,a) )}{2} \\ &\quad {}-\frac{\varGamma (\alpha +1)}{2 (e^{i \varphi }\eta (b,a) ) ^{\alpha }} \bigl[J_{a^{+}}^{\alpha }f \bigl(a+e^{i \varphi }\eta (b,a) \bigr) +J_{ (a+e^{i \varphi }\eta (b,a) ) ^{-}}^{\alpha }f(a) \bigr] \biggr\vert ,\bigl\vert f''(a) \bigr\vert , \bigl\vert f''(b) \bigr\vert \biggr) \\ & \quad {}+ \int _{0}^{1} L _{w(t)}\,dt \\ &=\frac{2(\alpha +1)}{ (e^{i \varphi }\eta (b,a) )^{2}} \biggl\vert \frac{f(a)+f (a+e^{i \varphi }\eta (b,a) )}{2} \\ &\quad {}-\frac{ \varGamma (\alpha +1)}{2 (e^{i \varphi }\eta (b,a) )^{\alpha }} \bigl[J_{a^{+}}^{\alpha }f \bigl(a+e^{i \varphi }\eta (b,a) \bigr) +J_{ (a+e^{i \varphi }\eta (b,a) )^{-}}^{\alpha }f(a) \bigr] \biggr\vert \\ &\quad{}- \biggl[\frac{\pi }{2}-\beta \biggl(\frac{1}{2},\alpha + \frac{5}{2} \biggr) - \beta \biggl(\frac{3}{2},\alpha + \frac{3}{2} \biggr) \biggr] \biggl( \bigl\vert f''(a ) \bigr\vert +\frac{1- \lambda }{\lambda } \bigl\vert f''(b) \bigr\vert \biggr). \end{aligned}$$

This leads to

$$\begin{aligned} & \biggl\vert \frac{f(a)+f (a+e^{i \varphi } \eta (b,a) )}{2}-\frac{ \varGamma (\alpha +1)}{2 (e^{i \varphi }\eta (b,a) )^{\alpha }} \bigl[J_{a^{+}}^{\alpha }f \bigl(a+e^{i \varphi }\eta (b,a) \bigr) +J_{ (a+e^{i \varphi }\eta (b,a) )^{-}}^{\alpha }f(a) \bigr] \biggr\vert \\ &\leq \frac{ (e^{i \varphi }\eta (b,a) )^{2}}{2(\alpha +1)} \biggl[\frac{\pi }{2}-\beta \biggl( \frac{1}{2},\alpha +\frac{5}{2} \biggr) -\beta \biggl( \frac{3}{2},\alpha +\frac{3}{2} \biggr) \biggr] \biggl( \bigl\vert f''(a) \bigr\vert +\frac{1- \lambda }{\lambda } \bigl\vert f''(b) \bigr\vert \biggr). \end{aligned}$$

Thus, the proof is completed. □

### Remark 13

In Corollary 11, if we choose

- $\eta (b,a)=b-a$ and $\varphi =0$, we get
  $$\begin{aligned} & \biggl\vert \frac{f(a)+f(b)}{2}-\frac{\varGamma (\alpha +1))}{2(b-a)^{\alpha }} \bigl[J_{a^{+}}^{\alpha }f(b)+J_{b^{-}}^{\alpha }f(a) \bigr] \biggr\vert \\ &\leq \frac{(b-a)^{2}}{2(\alpha +1)} \biggl[\frac{\pi }{2}-\beta \biggl( \frac{1}{2},\alpha +\frac{5}{2} \biggr) -\beta \biggl( \frac{3}{2}, \alpha +\frac{3}{2} \biggr) \biggr] \biggl( \bigl\vert f''(a) \bigr\vert +\frac{1- \lambda }{\lambda } \bigl\vert f''(b) \bigr\vert \biggr). \end{aligned}$$
- $\eta (b,a)=b-a$, $\varphi =0$, and $\lambda =\frac{1}{2}$, we get
  $$\begin{aligned} & \biggl\vert \frac{f(a)+f(b)}{2}-\frac{\varGamma (\alpha +1))}{2(b-a)^{\alpha }} \bigl[J_{a^{+}}^{\alpha }f(b)+J_{b^{-}}^{\alpha }f(a) \bigr] \biggr\vert \\ &\quad \leq \frac{(b-a)^{2}}{2(\alpha +1)} \biggl[\frac{\pi }{2}-\beta \biggl(\frac{1}{2}, \alpha +\frac{5}{2} \biggr) -\beta \biggl( \frac{3}{2},\alpha + \frac{3}{2} \biggr) \biggr] \bigl( \bigl\vert f''(a) \bigr\vert + \bigl\vert f''(b) \bigr\vert \bigr). \end{aligned}$$

### Corollary 12

*Under the assumptions of Theorem *12, *if* $|f''|$ *is* *h*-*convex*, *then we have*

$$\begin{aligned} & \biggl\vert \frac{f(a)+f (a+e^{i \varphi } \eta (b,a) )}{2}-\frac{ \varGamma (\alpha +1))}{2 (e^{i \varphi }\eta (b,a) )^{\alpha }} \bigl[J_{a^{+}}^{\alpha }f \bigl(a+e^{i \varphi }\eta (b,a) \bigr) +J_{ (a+e^{i \varphi }\eta (b,a) )^{-}}^{\alpha }f(a) \bigr] \biggr\vert \\ &\quad \leq \frac{ (e^{i \varphi }\eta (b,a) )^{2}}{2(\alpha +1)} \biggl( \int _{0}^{1} h(t) \bigl[1-(1-t)^{\alpha +1}-t^{\alpha +1} \bigr]\,dt \biggr) \biggl( \bigl\vert f''(a) \bigr\vert +\frac{1- \lambda }{\lambda } \bigl\vert f''(b) \bigr\vert \biggr). \end{aligned}$$

### Proof

Using (9) with $w(t)=1-(1-t)^{\alpha +1}-t^{\alpha +1}$, we obtain

$$\begin{aligned} &T_{F,w}(u_{1},u_{2},u_{3}) \\ &\quad =u_{1}- \biggl( \int _{0}^{1} h(t) \bigl[1-(1-t)^{\alpha +1}-t^{\alpha +1} \bigr]\,dt \biggr)u_{3} \\ &\qquad {}-\frac{1-\lambda }{\lambda } \biggl( \int _{0}^{1} h(1-t) \bigl[1-(1-t)^{\alpha +1}-t^{\alpha +1} \bigr]\,dt \biggr)u _{2} \\ &\quad =u_{1}- \biggl( \int _{0}^{1} h(t) \bigl[1-(1-t)^{\alpha +1}-t^{\alpha +1} \bigr]\,dt \biggr)u_{3} \\ &\qquad {}-\frac{1-\lambda }{\lambda } \biggl( \int _{0}^{1} h(t) \bigl[1-(1-t)^{\alpha +1}-t^{\alpha +1} \bigr]\,dt \biggr)u _{2} \\ &\quad =u_{1}- \biggl( \int _{0}^{1} h(t) \bigl[1-(1-t)^{\alpha +1}-t^{\alpha +1} \bigr]\,dt \biggr) \biggl(u_{2}+\frac{1-\lambda }{\lambda }u_{3} \biggr) \end{aligned}$$

for $u_{1}, u_{2}, u_{3}\in \mathbb{R}$, so Theorem 12 implies that

$$\begin{aligned} 0 &\geq T_{F,w} \biggl(\frac{2(\alpha +1)}{ (e^{i \varphi }\eta (b,a) ) ^{2}} \biggl\vert \frac{f(a)+f (a+e^{i \varphi } \eta (b,a) )}{2}\\ &\quad {}-\frac{ \varGamma (\alpha +1)}{2 (e^{i \varphi }\eta (b,a) )^{\alpha }} \bigl[J_{a^{+}}^{\alpha }f \bigl(a+e^{i \varphi }\eta (b,a) \bigr) +J_{ (a+e^{i \varphi }\eta (b,a) )^{-}}^{\alpha }f(a) \bigr] \biggr\vert ,\bigl\vert f''(a) \bigr\vert , \bigl\vert f''(b) \bigr\vert \biggr) \\ & \quad {}+ \int _{0}^{1} L _{w(t)}\,dt \\ &=\frac{2(\alpha +1)}{ (e^{i \varphi }\eta (b,a) )^{2}} \biggl\vert \frac{f(a)+f (a+e^{i \varphi } \eta (b,a) )}{2}\\ &\quad {}-\frac{ \varGamma (\alpha +1)}{2 (e^{i \varphi }\eta (b,a) )^{\alpha }} \bigl[J_{a^{+}}^{\alpha }f \bigl(a+e^{i \varphi }\eta (b,a) \bigr) +J_{ (a+e^{i \varphi }\eta (b,a) )^{-}}^{\alpha }f(a) \bigr] \biggr\vert \\ &\quad{}- \biggl( \int _{0}^{1} h(t) \bigl[1-(1-t)^{\alpha +1}-t^{\alpha +1} \bigr]\,dt \biggr) \biggl( \bigl\vert f''(a) \bigr\vert +\frac{1- \lambda }{\lambda } \bigl\vert f''(b) \bigr\vert \biggr), \end{aligned}$$

which can be written as

$$\begin{aligned} & \biggl\vert \frac{f(a)+f (a+e^{i \varphi } \eta (b,a) )}{2}-\frac{ \varGamma (\alpha +1)}{2 (e^{i \varphi }\eta (b,a) )^{\alpha }} \bigl[J_{a^{+}}^{\alpha }f \bigl(a+e^{i \varphi }\eta (b,a) \bigr) +J_{ (a+e^{i \varphi }\eta (b,a) )^{-}}^{\alpha }f(a) \bigr] \biggr\vert \\ &\quad \leq \frac{ (e^{i \varphi }\eta (b,a) )^{2}}{2(\alpha +1)} \biggl( \int _{0}^{1} h(t) \bigl[1-(1-t)^{\alpha +1}-t^{\alpha +1} \bigr]\,dt \biggr) \biggl( \bigl\vert f''(a) \bigr\vert +\frac{1- \lambda }{\lambda } \bigl\vert f''(b) \bigr\vert \biggr). \end{aligned}$$

Thus, the proof is done. □

### Theorem 13

*Let* $f: [0,b]\to \mathbb{R}$ *be a differentiable mapping and* $|f''|^{{\frac{p}{p-1}}}$ *is measurable*, *decreasing*, $\lambda _{\varphi }$-*preinvex function on* $[0,b]$ *for* $\eta (b,a)>0$ *and* $0\leq a< b$. *Suppose that* $|f''|^{{\frac{p}{p-1}}}$ *is* *F*-*convex on* $[a,b]$, *for some* $F\in \mathcal{F}$ *and* $|f''|\in L^{{\frac{p}{p-1}}}(a,b)$, $p>1$. *Then we have*

20 $$\begin{aligned} &T_{F,1} \bigl(H_{1}(f,p), \bigl\vert f''(a) \bigr\vert ^{\frac{p}{p-1}}, \bigl\vert f''(b) \bigr\vert ^{\frac{p}{p-1}} \bigr)\leq 0, \end{aligned}$$

*where*

$$\begin{aligned} H_{1}(f,p) &= \biggl(\frac{2^{\alpha +1}(\alpha +1)}{ (2^{\alpha }-1 ) (e^{i \varphi }\eta (b,a) )^{2}} \biggr)^{\frac{p}{p-1}} \biggl\vert \frac{f(a)+f (a+e^{i \varphi } \eta (b,a) )}{2} \\ &\quad{}-\frac{ \varGamma (\alpha +1)}{2 (e^{i \varphi }\eta (b,a) )^{\alpha }} \bigl[J_{a^{+}}^{\alpha }f \bigl(a+e^{i \varphi }\eta (b,a) \bigr) +J_{ (a+e^{i \varphi }\eta (b,a) )^{-}}^{\alpha }f(a) \bigr] \biggr\vert ^{\frac{p}{p-1}}. \end{aligned}$$

### Proof

Since $|f''|^{\frac{p}{p-1}}$ is *F*-convex, we have

$$\begin{aligned} F \bigl( \bigl\vert f'' \bigl(a+(1-t)e^{i \varphi } \eta (b,a) \bigr) \bigr\vert ^{\frac{p}{p-1}}, \bigl\vert f''(a) \bigr\vert ^{\frac{p}{p-1}}, \bigl\vert f''(b) \bigr\vert ^{\frac{p}{p-1}},t \bigr) \leq 0,\quad t\in [0,1]. \end{aligned}$$

Using (A2) with $w(t)=1$, we have

$$\begin{aligned} T_{F,1} \biggl( \int _{0}^{1} \bigl\vert f \bigl(a+(1-t)e^{i \varphi } \eta (b,a) \bigr) \bigr\vert ^{\frac{p}{p-1}} \,dt, \bigl\vert f''(a) \bigr\vert ^{\frac{p}{p-1}}, \bigl\vert f''(b) \bigr\vert ^{\frac{p}{p-1}} \biggr) \leq 0, \quad t\in [0,1]. \end{aligned}$$

Using Lemma 2, Lemma 3 and the Hölder inequality, we get

$$\begin{aligned} & \biggl\vert \frac{f(a)+f (a+e^{i \varphi } \eta (b,a) )}{2}-\frac{ \varGamma (\alpha +1)}{2 (e^{i \varphi }\eta (b,a) )^{\alpha }} \bigl[J_{a^{+}}^{\alpha }f \bigl(a+e^{i \varphi }\eta (b,a) \bigr) +J_{ (a+e^{i \varphi }\eta (b,a) )^{-}}^{\alpha }f(a) \bigr] \biggr\vert \\ &\quad =\frac{ (e^{i \varphi }\eta (b,a) )^{2}}{2(\alpha +1)} \int _{0}^{1} \bigl[1-(1-t)^{\alpha +1}-t^{\alpha +1} \bigr] \bigl\vert f'' \bigl(a+e^{i \varphi }\eta (b,a) \bigr) \bigr\vert \,dt \\ &\quad \leq \frac{ (e^{i \varphi }\eta (b,a) )^{2}}{2(\alpha +1)} \biggl( \int _{0}^{1} \bigl[1-(1-t)^{\alpha +1}-t^{\alpha +1} \bigr] ^{p} \,dt \biggr)^{\frac{1}{p}}\\ &\qquad {}\times \biggl( \int _{0}^{1} \bigl\vert f'' \bigl(a+(1-t)e ^{i \varphi }\eta (b,a) \bigr) \bigr\vert ^{\frac{p}{p-1}}\,dt \biggr) ^{\frac{p-1}{p}} \\ &\quad =\frac{ (e^{i \varphi }\eta (b,a) )^{2}}{2(\alpha +1)} \biggl(1-\frac{1}{2^{\alpha }} \biggr) \biggl( \int _{0}^{1} \bigl\vert f'' \bigl(a+(1-t)e^{i \varphi }\eta (b,a) \bigr) \bigr\vert ^{\frac{p}{p-1}}\,dt \biggr)^{\frac{p-1}{p}} \end{aligned}$$

or, equivalently,

$$\begin{aligned} & \biggl(\frac{2^{\alpha +1}(\alpha +1)}{ (2^{\alpha }-1 ) (e^{i \varphi }\eta (b,a) )^{2}} \biggr)^{\frac{p}{p-1}}\biggl\vert \frac{f(a)+f (a+e^{i \varphi } \eta (b,a) )}{2} \\ &\qquad{} -\frac{ \varGamma (\alpha +1)}{2 (e^{i \varphi }\eta (b,a) )^{\alpha }} \bigl[J_{a^{+}}^{\alpha }f \bigl(a+e^{i \varphi }\eta (b,a) \bigr) +J_{ (a+e^{i \varphi }\eta (b,a) )^{-}}^{\alpha }f(a) \bigr] \biggr\vert ^{\frac{p}{p-1}} \\ &\quad \leq \int _{0}^{1} \bigl\vert f'' \bigl(a+(1-t)e^{i \varphi }\eta (b,a) \bigr) \bigr\vert ^{\frac{p}{p-1}}\,dt. \end{aligned}$$

Because $T_{F,1}$ is nondecreasing with respect to the first variable, we get

$$\begin{aligned} &T_{F,1} \bigl(H_{1}(f,p), \bigl\vert f''(a) \bigr\vert ^{{\frac{p}{p-1}}}, \bigl\vert f''(b) \bigr\vert ^{{\frac{p}{p-1}}} \bigr)\leq 0. \end{aligned}$$

Thus, the proof is completed. □

### Remark 14

If we choose $\eta (b,a)=b-a$ and $\varphi =0$ in Theorem 13, we get

$$\begin{aligned} &T_{F,1} \biggl( \biggl(\frac{2^{\alpha +1}(\alpha +1)}{ (2^{ \alpha }-1 ) (b-a)^{2}} \biggr)^{\frac{p}{p-1}} \biggl\vert \frac{f(a)+f(b)}{2} -\frac{ \varGamma (\alpha +1))}{2(b-a)^{\alpha }} \bigl[J_{a^{+}}^{\alpha }f(b)+J _{b^{-}}^{\alpha }f(a) \bigr] \biggr\vert , \\ &\quad \bigl\vert f''(a) \bigr\vert ,\bigl\vert f''(b) \bigr\vert \biggr) \leq 0. \end{aligned}$$

### Corollary 13

*Under the assumptions of Theorem *13, *if* $|f''|^{ \frac{p}{p-1}}$ *is* *ε*-*convex*, *then*

$$\begin{aligned} & \biggl\vert \frac{f(a)+f (a+e^{i \varphi } \eta (b,a) )}{2}-\frac{ \varGamma (\alpha +1))}{2 (e^{i \varphi }\eta (b,a) )^{\alpha }} \bigl[J_{a^{+}}^{\alpha }f \bigl(a+e^{i \varphi }\eta (b,a) \bigr) +J_{ (a+e^{i \varphi }\eta (b,a) )^{-}}^{\alpha }f(a) \bigr] \biggr\vert \\ &\quad \leq \biggl(\frac{ (2^{\alpha }-1 ) (e^{i \varphi } \eta (b,a) )^{2}}{2^{\alpha +1}(\alpha +1)} \biggr) \biggl(\frac{ \vert f''(a) \vert ^{\frac{p}{p-1}}+ \vert f''(b) \vert ^{ \frac{p}{p-1}}}{2}+\varepsilon \biggr)^{\frac{p-1}{p}}. \end{aligned}$$

### Proof

Using (12), (13), by Theorem 13, we have

$$\begin{aligned} 0 &\geq T_{F,1} \bigl(H_{1}(f,p), \bigl\vert f''(a) \bigr\vert ^{\frac{p}{p-1}}, \bigl\vert f''(b) \bigr\vert ^{\frac{p}{p-1}} \bigr) \\ &=H_{1}(f,p)-\frac{ \vert f''(a) \vert ^{\frac{p}{p-1}}+ \vert f''(b) \vert ^{\frac{p}{p-1}}}{2} -\varepsilon . \end{aligned}$$

This leads to

$$\begin{aligned} & \biggl\vert \frac{f(a)+f (a+e^{i \varphi } \eta (b,a) )}{2}-\frac{ \varGamma (\alpha +1))}{2 (e^{i \varphi }\eta (b,a) )^{\alpha }} \bigl[J_{a^{+}}^{\alpha }f \bigl(a+e^{i \varphi }\eta (b,a) \bigr) +J_{ (a+e^{i \varphi }\eta (b,a) )^{-}}^{\alpha }f(a) \bigr] \biggr\vert \\ &\quad \leq \biggl(\frac{ (2^{\alpha }-1 ) (e^{i \varphi } \eta (b,a) )^{2}}{2^{\alpha +1}(\alpha +1)} \biggr) \biggl(\frac{ \vert f''(a) \vert ^{\frac{p}{p-1}} + \vert f''(b) \vert ^{ \frac{p}{p-1}}}{2}+\varepsilon \biggr)^{\frac{p-1}{p}}. \end{aligned}$$

This completes the proof. □

### Remark 15

In Corollary 13, if we choose

- $\eta (b,a)=b-a$ and $\varphi =0$, we get
  $$\begin{aligned} & \biggl\vert \frac{f(a)+f(b)}{2}-\frac{\varGamma (\alpha +1))}{2(b-a)^{\alpha }} \bigl[J_{a^{+}}^{\alpha }f(b)+J_{b^{-}}^{\alpha }f(a) \bigr] \biggr\vert \\ &\quad \leq \biggl(\frac{ (2^{\alpha }-1 )(b-a)^{2}}{2^{\alpha +1}( \alpha +1)} \biggr) \biggl( \frac{ \vert f''(a) \vert ^{\frac{p}{p-1}} + \vert f''(b) \vert ^{\frac{p}{p-1}}}{2}+\varepsilon \biggr)^{ \frac{p-1}{p}}. \end{aligned}$$
- $\eta (b,a)=b-a$, $\varphi =0$, and $\varepsilon =0$, we get
  $$\begin{aligned} & \biggl\vert \frac{f(a)+f(b)}{2}-\frac{\varGamma (\alpha +1))}{2(b-a)^{\alpha }} \bigl[J_{a^{+}}^{\alpha }f(b)+J_{b^{-}}^{\alpha }f(a) \bigr] \biggr\vert \\ &\quad \leq \biggl(\frac{ (2^{\alpha }-1 )(b-a)^{2}}{2^{\alpha +1}( \alpha +1)} \biggr) \biggl( \frac{ \vert f''(a) \vert ^{\frac{p}{p-1}}+ \vert f''(b) \vert ^{\frac{p}{p-1}}}{2} \biggr)^{\frac{p-1}{p}}. \end{aligned}$$

### Corollary 14

*Under the assumptions of Theorem *13. *If* $|f''|^{ \frac{p-1}{p}}$ *is* $\lambda _{\varphi }$-*preinvex*, *we have*

$$\begin{aligned} & \biggl\vert \frac{f(a)+f (a+e^{i \varphi } \eta (b,a) )}{2}-\frac{ \varGamma (\alpha +1))}{2 (e^{i \varphi }\eta (b,a) )^{\alpha }} \bigl[J_{a^{+}}^{\alpha }f \bigl(a+e^{i \varphi }\eta (b,a) \bigr) +J_{ (a+e^{i \varphi }\eta (b,a) )^{-}}^{\alpha }f(a) \bigr] \biggr\vert \\ &\quad \leq \biggl(\frac{ (2^{\alpha }-1 ) (e^{i \varphi } \eta (b,a) )^{2}}{2^{\alpha +1}(\alpha +1)} \biggr) \biggl[\frac{ \pi }{4} \biggl( \bigl\vert f''(a) \bigr\vert ^{\frac{p-1}{p}} + \frac{1-\lambda }{\lambda } \bigl\vert f''(b) \bigr\vert ^{\frac{p-1}{p}} \biggr) \biggr]^{ \frac{p-1}{p}}. \end{aligned}$$

### Proof

Using (7), (14), by Theorem 13, we have

$$\begin{aligned} 0 &\geq T_{F,1} \bigl(H_{1}(f,p), \bigl\vert f''(a) \bigr\vert ^{\frac{p-1}{p}}, \bigl\vert f''(b) \bigr\vert ^{\frac{p-1}{p}} \bigr) \\ &= \biggl(\frac{2^{\alpha +1}(\alpha +1)}{ (2^{\alpha }-1 ) (e^{i \varphi }\eta (b,a) )^{2}} \biggr)^{\frac{p}{p-1}}\biggl\vert \frac{f(a)+f (a+e^{i \varphi } \eta (b,a) )}{2} \\ &\quad{} -\frac{ \varGamma (\alpha +1)}{2 (e^{i \varphi }\eta (b,a) )^{\alpha }} \bigl[J_{a^{+}}^{\alpha }f \bigl(a+e^{i \varphi }\eta (b,a) \bigr) +J_{ (a+e^{i \varphi }\eta (b,a) )^{-}}^{\alpha }f(a) \bigr] \biggr\vert ^{\frac{p}{p-1}} \\ &\quad{}-\frac{\pi }{4} \biggl( \bigl\vert f''(a) \bigr\vert ^{\frac{p-1}{p}} +\frac{1- \lambda }{\lambda } \bigl\vert f''(b) \bigr\vert ^{\frac{p-1}{p}} \biggr), \end{aligned}$$

that is,

$$\begin{aligned} & \biggl\vert \frac{f(a)+f (a+e^{i \varphi } \eta (b,a) )}{2}-\frac{ \varGamma (\alpha +1)}{2 (e^{i \varphi }\eta (b,a) )^{\alpha }} \bigl[J_{a^{+}}^{\alpha }f \bigl(a+e^{i \varphi }\eta (b,a) \bigr) +J_{ (a+e^{i \varphi }\eta (b,a) )^{-}}^{\alpha }f(a) \bigr] \biggr\vert \\ &\quad \leq \biggl(\frac{ (2^{\alpha }-1 ) (e^{i \varphi } \eta (b,a) )^{2}}{2^{\alpha +1}(\alpha +1)} \biggr) \biggl[\frac{ \pi }{4} \biggl( \bigl\vert f''(a) \bigr\vert ^{\frac{p-1}{p}} + \frac{1-\lambda }{\lambda } \bigl\vert f''(b) \bigr\vert ^{\frac{p-1}{p}} \biggr) \biggr]^{ \frac{p-1}{p}}. \end{aligned}$$

This proves Corollary 14. □

### Remark 16

In Corollary 14, if we choose

- $\eta (b,a)=b-a$ and $\varphi =0$, we get
  $$\begin{aligned} & \biggl\vert \frac{f(a)+f(b)}{2}-\frac{\varGamma (\alpha +1))}{2(b-a)^{\alpha }} \bigl[J_{a^{+}}^{\alpha }f(b)+J_{b^{-}}^{\alpha }f(a) \bigr] \biggr\vert \\ &\quad \leq \biggl(\frac{ (2^{\alpha }-1 )(b-a)^{2}}{2^{\alpha +1}( \alpha +1)} \biggr) \biggl[ \frac{\pi }{4} \biggl( \bigl\vert f''(a) \bigr\vert ^{\frac{p-1}{p}} +\frac{1-\lambda }{\lambda } \bigl\vert f''(b) \bigr\vert ^{\frac{p-1}{p}} \biggr) \biggr]^{\frac{p-1}{p}}. \end{aligned}$$
- $\eta (b,a)=b-a$, $\varphi =0$, and $\lambda =\frac{1}{2}$, we get
  $$\begin{aligned} & \biggl\vert \frac{f(a)+f(b)}{2}-\frac{\varGamma (\alpha +1))}{2(b-a)^{\alpha }} \bigl[J_{a^{+}}^{\alpha }f(b)+J_{b^{-}}^{\alpha }f(a) \bigr] \biggr\vert \\ &\quad \leq \biggl(\frac{ (2^{\alpha }-1 )(b-a)^{2}}{2^{\alpha +1}( \alpha +1)} \biggr) \biggl[ \frac{\pi \vert f''(a) \vert ^{ \frac{p-1}{p}} +\pi \vert f''(b) \vert ^{\frac{p-1}{p}}}{4} \biggr] ^{\frac{p-1}{p}}. \end{aligned}$$

### Corollary 15

*Under the assumptions of Theorem *13. *If* $|f''|^{ \frac{p}{p-1}}$ *is* *h*-*convex*, *then*

$$\begin{aligned} & \biggl\vert \frac{f(a)+f (a+e^{i \varphi } \eta (b,a) )}{2}-\frac{ \varGamma (\alpha +1))}{2 (e^{i \varphi }\eta (b,a) )^{\alpha }} \bigl[J_{a^{+}}^{\alpha }f \bigl(a+e^{i \varphi }\eta (b,a) \bigr) +J_{ (a+e^{i \varphi }\eta (b,a) )^{-}}^{\alpha }f(a) \bigr] \biggr\vert \\ &\quad \leq \biggl(\frac{ (2^{\alpha }-1 ) (e^{i \varphi } \eta (b,a) )^{2}}{2^{\alpha +1}(\alpha +1)} \biggr) \biggl( \int _{0}^{1} h(t) \biggr)^{\frac{p-1}{p}} \biggl( \bigl\vert f''(a) \bigr\vert ^{ \frac{p-1}{p}} + \frac{1-\lambda }{\lambda } \bigl\vert f''(b) \bigr\vert ^{ \frac{p-1}{p}} \biggr). \end{aligned}$$

### Proof

Using (9) and by Theorem 13, it can be proved easily. It is omitted. □

### Theorem 14

*Let* $f: [0,b]\to \mathbb{R}$ *be a differentiable mapping and* $|f''|^{{\frac{p}{p-1}}}$ *is measurable*, *decreasing*, $\lambda _{\varphi }$-*preinvex function on* $[0,b]$ *for* $\eta (b,a)>0$ *and* $0\leq a< b$. *Suppose that* $|f''|^{{\frac{p}{p-1}}}$ *is* *F*-*convex on* $[a,b]$, *for some* $F\in \mathcal{F}$ *and* $|f''|\in L^{{\frac{p}{p-1}}}(a,b)$, $p>1$. *Then we have*

21 $$\begin{aligned} &T_{F,1} \bigl(H_{2}(f,p), \bigl\vert f''(a) \bigr\vert ^{\frac{p}{p-1}}, \bigl\vert f''(b) \bigr\vert ^{\frac{p}{p-1}} \bigr)+ \int _{0}^{1} L_{w(t)}\,dt \leq 0, \end{aligned}$$

*where*

$$\begin{aligned} H_{2}(f,p) & = \biggl(\frac{2(\alpha +1)}{ (e^{i \varphi }\eta (b,a) ) ^{2}} \biggr)^{\frac{p}{p-1}} \biggl(\frac{2^{\alpha }}{2^{\alpha }-1} \biggr) ^{\frac{1}{p-1}} \biggl\vert \frac{f(a)+f (a+e^{i \varphi } \eta (b,a) )}{2} \\ &\quad{}-\frac{ \varGamma (\alpha +1)}{2 (e^{i \varphi }\eta (b,a) )^{\alpha }} \bigl[J_{a^{+}}^{\alpha }f \bigl(a+e^{i \varphi }\eta (b,a) \bigr) +J_{ (a+e^{i \varphi }\eta (b,a) )^{-}}^{\alpha }f(a) \bigr] \biggr\vert ^{\frac{p}{p-1}} \end{aligned}$$

*for* $w(t)=1-(1-t)^{\alpha +1}-t^{\alpha +1}$.

### Proof

Since $|f''|^{\frac{p}{p-1}}$ is *F*-convex, we have

$$\begin{aligned} F \bigl( \bigl\vert f'' \bigl(a+(1-t)e^{i \varphi } \eta (b,a) \bigr) \bigr\vert ^{\frac{p}{p-1}}, \bigl\vert f''(a) \bigr\vert ^{\frac{p}{p-1}}, \bigl\vert f''(b) \bigr\vert ^{\frac{p}{p-1}},t \bigr) \leq 0,\quad t\in [0,1]. \end{aligned}$$

Using (A3) with $w(t)=1-(1-t)^{\alpha +1}-t^{\alpha +1}$, we obtain

$$\begin{aligned} &F \bigl(w(t) \bigl\vert f'' \bigl(a+(1-t)e^{i \varphi } \eta (b,a) \bigr) \bigr\vert ^{\frac{p}{p-1}}, w(t) \bigl\vert f''(a) \bigr\vert ^{\frac{p}{p-1}},w(t) \bigl\vert f''(b) \bigr\vert ^{\frac{p}{p-1}},t \bigr) +L_{w(t)}\leq 0,\\ &\quad t\in [0,1]. \end{aligned}$$

Integrating over $[0,1]$ and using axiom (A2), we obtain

$$\begin{aligned} &T_{F,w} \biggl( \int _{0}^{1} w(t) \bigl\vert f'' \bigl(a+(1-t)e^{i \varphi } \eta (b,a) \bigr) \bigr\vert ^{\frac{p}{p-1}}\,dt, \bigl\vert f''(a) \bigr\vert ^{\frac{p}{p-1}}, \bigl\vert f''(b) \bigr\vert ^{\frac{p}{p-1}} \biggr)+ \int _{0}^{1} L_{w(t)}\,dt\leq 0,\\ &\quad t\in [0,1]. \end{aligned}$$

Using Lemma 2 and the power mean inequality, we get

$$\begin{aligned} & \biggl\vert \frac{f(a)+f (a+e^{i \varphi } \eta (b,a) )}{2}-\frac{ \varGamma (\alpha +1)}{2 (e^{i \varphi }\eta (b,a) )^{\alpha }} \bigl[J_{a^{+}}^{\alpha }f \bigl(a+e^{i \varphi }\eta (b,a) \bigr) +J_{ (a+e^{i \varphi }\eta (b,a) )^{-}}^{\alpha }f(a) \bigr] \biggr\vert \\ &\quad =\frac{e^{i \varphi }\eta (b,a)}{2} \int _{0}^{1} \bigl[1-(1-t)^{ \alpha +1}-t^{\alpha +1} \bigr] \bigl\vert f'' \bigl(a+(1-t)e^{i \varphi } \eta (b,a) \bigr) \bigr\vert \,dt \\ &\quad \leq \frac{ (e^{i \varphi }\eta (b,a) )^{2}}{2(\alpha +1)} \biggl( \int _{0}^{1} \bigl[1-(1-t)^{\alpha +1}-t^{\alpha +1} \bigr]\,dt \biggr) ^{\frac{1}{p}}\\ &\qquad {}\times \biggl( \int _{0}^{1} w(t) \bigl\vert f' \bigl(a+(1-t)e^{i \varphi }\eta (b,a) \bigr) \bigr\vert ^{\frac{p}{p-1}}\,dt \biggr)^{ \frac{p-1}{p}} \\ &\quad =\frac{ (e^{i \varphi }\eta (b,a) )^{2}}{2(\alpha +1)} \biggl(1-\frac{1}{2^{\alpha }} \biggr)^{\frac{1}{p}} \biggl( \int _{0} ^{1} w(t) \bigl\vert f' \bigl(a+(1-t)e^{i \varphi }\eta (b,a) \bigr) \bigr\vert ^{\frac{p}{p-1}}\,dt \biggr) ^{\frac{p-1}{p}} \end{aligned}$$

or, equivalently,

$$\begin{aligned} & \biggl(\frac{2(\alpha +1)}{ (e^{i \varphi }\eta (b,a) ) ^{2}} \biggr)^{\frac{p}{p-1}} \biggl(\frac{2^{\alpha }}{2^{\alpha }-1} \biggr) ^{\frac{1}{p-1}} \biggl\vert \frac{f(a)+f (a+e^{i \varphi } \eta (b,a) )}{2} \\ &\qquad{}-\frac{ \varGamma (\alpha +1)}{2 (e^{i \varphi }\eta (b,a) )^{\alpha }} \bigl[J_{a^{+}}^{\alpha }f \bigl(a+e^{i \varphi }\eta (b,a) \bigr) +J_{ (a+e^{i \varphi }\eta (b,a) )^{-}}^{\alpha }f(a) \bigr] \biggr\vert ^{\frac{p}{p-1}} \\ &\quad \leq \int _{0}^{1} w(t) \bigl\vert f'' \bigl(a+(1-t)e^{i \varphi }\eta (b,a) \bigr) \bigr\vert ^{\frac{p}{p-1}}\,dt. \end{aligned}$$

Since $T_{F,w}$ is nondecreasing with respect to the first variable, we have

$$\begin{aligned} &T_{F,w} \bigl(H_{2}(f,p), \bigl\vert f''(a) \bigr\vert ^{{\frac{p}{p-1}}}, \bigl\vert f''(b) \bigr\vert ^{{\frac{p}{p-1}}} \bigr)+ \int _{0}^{1} L_{w(t)}\,dt \leq 0. \end{aligned}$$

This completes the proof. □

### Remark 17

Taking $\eta (b,a)=b-a$ and $\varphi =0$ in Theorem 14, we get

$$\begin{aligned} &T_{F,w} \biggl( \biggl(\frac{2(\alpha +1)}{ (e^{i \varphi }\eta (b,a) ) ^{2}} \biggr)^{\frac{p}{p-1}} \biggl(\frac{2^{\alpha }}{2^{\alpha }-1} \biggr) ^{\frac{1}{p-1}} \biggl\vert \frac{f(a)+f(b)}{2}- \frac{\varGamma (\alpha +1))}{2(b-a)^{ \alpha }} \bigl[J_{a^{+}}^{\alpha }f(b)+J_{b^{-}}^{\alpha }f(a) \bigr] \biggr\vert , \\ &\quad\bigl\vert f'(a) \bigr\vert , \bigl\vert f'(b) \bigr\vert \biggr)+ \int _{0}^{1} L_{w(t)}\,dt\leq 0. \end{aligned}$$

### Corollary 16

*Under the assumptions of Theorem *14, *if* $|f''|^{ \frac{p}{p-1}}$ *is* *ε*-*convex*, *we have*

$$\begin{aligned} & \biggl\vert \frac{f(a)+f (a+e^{i \varphi } \eta (b,a) )}{2}-\frac{ \varGamma (\alpha +1))}{2 (e^{i \varphi }\eta (b,a) )^{\alpha }} \bigl[J_{a^{+}}^{\alpha }f \bigl(a+e^{i \varphi }\eta (b,a) \bigr) +J_{ (a+e^{i \varphi }\eta (b,a) )^{-}}^{\alpha }f(a) \bigr] \biggr\vert \\ &\quad \leq \biggl(\frac{ (e^{i \varphi }\eta (b,a) )^{2}}{2( \alpha +1)} \biggr) \biggl(\frac{2^{\alpha }-1}{2^{\alpha }} \biggr) ^{\frac{1}{p}} \biggl[\frac{\alpha }{2(\alpha +2)} \bigl( \bigl\vert f''(a) \bigr\vert ^{\frac{p}{p-1}} + \bigl\vert f''(b) \bigr\vert ^{\frac{p}{p-1}} \bigr)+2 \varepsilon \biggr]^{\frac{p-1}{p}}. \end{aligned}$$

### Proof

Using (17), (18) and by Theorem 13, we obtain

$$\begin{aligned} & \biggl\vert \frac{f(a)+f (a+e^{i \varphi } \eta (b,a) )}{2}-\frac{ \varGamma (\alpha +1))}{2 (e^{i \varphi }\eta (b,a) )^{\alpha }} \bigl[J_{a^{+}}^{\alpha }f \bigl(a+e^{i \varphi }\eta (b,a) \bigr) +J_{ (a+e^{i \varphi }\eta (b,a) )^{-}}^{\alpha }f(a) \bigr] \biggr\vert \\ &\quad \leq \biggl(\frac{ (e^{i \varphi }\eta (b,a) )^{2}}{2( \alpha +1)} \biggr) \biggl(\frac{2^{\alpha }-1}{2^{\alpha }} \biggr) ^{\frac{1}{p}} \biggl[\frac{\alpha }{2(\alpha +2)} \bigl( \bigl\vert f''(a) \bigr\vert ^{\frac{p}{p-1}} + \bigl\vert f''(b) \bigr\vert ^{\frac{p}{p-1}} \bigr)+2 \varepsilon \biggr]^{\frac{p-1}{p}}. \end{aligned}$$

This completes the proof. □

### Remark 18

In Corollary 16, if we choose

- $\eta (b,a)=b-a$ and $\varphi =0$, we get
  $$\begin{aligned} & \biggl\vert \frac{f(a)+f(b)}{2}-\frac{\varGamma (\alpha +1))}{2(b-a)^{\alpha }} \bigl[J_{a^{+}}^{\alpha }f(b)+J_{b^{-}}^{\alpha }f(a) \bigr] \biggr\vert \\ &\quad \leq \biggl(\frac{(b-a)^{2}}{2(\alpha +1)} \biggr) \biggl(\frac{2^{ \alpha }-1}{2^{\alpha }} \biggr)^{\frac{1}{p}} \biggl[\frac{\alpha }{2( \alpha +2)} \bigl( \bigl\vert f''(a) \bigr\vert ^{\frac{p}{p-1}} + \bigl\vert f''(b) \bigr\vert ^{\frac{p}{p-1}} \bigr)+2\varepsilon \biggr]^{\frac{p-1}{p}}. \end{aligned}$$
- $\eta (b,a)=b-a$, $\varphi =0$, and $\varepsilon =0$, we get
  $$\begin{aligned} & \biggl\vert \frac{f(a)+f(b)}{2}-\frac{\varGamma (\alpha +1))}{2(b-a)^{\alpha }} \bigl[J_{a^{+}}^{\alpha }f(b)+J_{b^{-}}^{\alpha }f(a) \bigr] \biggr\vert \\ &\quad \leq \biggl(\frac{(b-a)^{2}}{2(\alpha +1)} \biggr) \biggl( \frac{2^{ \alpha }-1}{2^{\alpha }} \biggr)^{\frac{1}{p}} \biggl[\frac{\alpha }{2( \alpha +2)} \bigl( \bigl\vert f''(a) \bigr\vert ^{\frac{p}{p-1}} + \bigl\vert f''(b) \bigr\vert ^{\frac{p}{p-1}} \bigr) \biggr]^{\frac{p-1}{p}}. \end{aligned}$$

### Corollary 17

*Under the assumptions of Theorem *14. *If* $|f''|^{ \frac{p-1}{p}}$ *is* $\lambda _{\varphi }$-*preinvex*, *then*

$$\begin{aligned} & \biggl\vert \frac{f(a)+f (a+e^{i \varphi } \eta (b,a) )}{2}-\frac{ \varGamma (\alpha +1))}{2 (e^{i \varphi }\eta (b,a) )^{\alpha }} \bigl[J_{a^{+}}^{\alpha }f \bigl(a+e^{i \varphi }\eta (b,a) \bigr) +J_{ (a+e^{i \varphi }\eta (b,a) )^{-}}^{\alpha }f(a) \bigr] \biggr\vert \\ &\quad \leq \biggl(\frac{ (e^{i \varphi }\eta (b,a) )^{2}}{2( \alpha +1)} \biggr) \biggl(\frac{2^{\alpha }-1}{2^{\alpha }} \biggr) ^{\frac{1}{p}}\\ &\qquad {}\times \biggl( \biggl[\frac{\pi }{2}-\beta \biggl(\frac{1}{2}, \alpha +\frac{5}{2} \biggr) -\beta \biggl(\frac{3}{2},\alpha + \frac{3}{2} \biggr) \biggr] \biggl( \bigl\vert f''(a) \bigr\vert ^{ \frac{p}{p-1}}+\frac{1-\lambda }{\lambda } \bigl\vert f''(b) \bigr\vert ^{ \frac{p}{p-1}} \biggr) \biggr)^{\frac{p-1}{p}}. \end{aligned}$$

### Proof

Using (19), by Theorem 14, we have

$$\begin{aligned} 0 &\geq T_{F,w} \bigl(H_{2}(f,p), \bigl\vert f''(a) \bigr\vert ^{\frac{p-1}{p}}, \bigl\vert f''(b) \bigr\vert ^{\frac{p-1}{p}} \bigr) \\ &= \biggl(\frac{2(\alpha +1)}{ (e^{i \varphi }\eta (b,a) ) ^{2}} \biggr)^{\frac{p}{p-1}} \biggl(\frac{2^{\alpha }}{2^{\alpha }-1} \biggr) ^{\frac{1}{p-1}}\biggl\vert \frac{f(a)+f (a+e^{i \varphi } \eta (b,a) )}{2} \\ &\quad{}-\frac{ \varGamma (\alpha +1)}{2 (e^{i \varphi }\eta (b,a) )^{\alpha }} \bigl[J_{a^{+}}^{\alpha }f \bigl(a+e^{i \varphi }\eta (b,a) \bigr) +J_{ (a+e^{i \varphi }\eta (b,a) )^{-}}^{\alpha }f(a) \bigr] \biggr\vert ^{\frac{p}{p-1}} \\ &\quad{}- \biggl[\frac{\pi }{2}-\beta \biggl(\frac{1}{2},\alpha + \frac{5}{2} \biggr) - \beta \biggl(\frac{3}{2},\alpha + \frac{3}{2} \biggr) \biggr] \biggl( \bigl\vert f''(a) \bigr\vert ^{\frac{p-1}{p}} +\frac{1-\lambda }{\lambda } \bigl\vert f''(b) \bigr\vert ^{\frac{p-1}{p}} \biggr). \end{aligned}$$

This leads to

$$\begin{aligned} & \biggl\vert \frac{f(a)+f (a+e^{i \varphi } \eta (b,a) )}{2}-\frac{ \varGamma (\alpha +1)}{2 (e^{i \varphi }\eta (b,a) )^{\alpha }} \bigl[J_{a^{+}}^{\alpha }f \bigl(a+e^{i \varphi }\eta (b,a) \bigr) +J_{ (a+e^{i \varphi }\eta (b,a) )^{-}}^{\alpha }f(a) \bigr] \biggr\vert \\ &\quad \leq \biggl(\frac{ (e^{i \varphi }\eta (b,a) )^{2}}{2( \alpha +1)} \biggr) \biggl(\frac{2^{\alpha }-1}{2^{\alpha }} \biggr) ^{\frac{1}{p}}\\ &\qquad {}\times \biggl( \biggl[\frac{\pi }{2}-\beta \biggl(\frac{1}{2}, \alpha +\frac{5}{2} \biggr) -\beta \biggl(\frac{3}{2},\alpha + \frac{3}{2} \biggr) \biggr] \biggl( \bigl\vert f''(a) \bigr\vert ^{ \frac{p}{p-1}}+\frac{1-\lambda }{\lambda } \bigl\vert f''(b) \bigr\vert ^{ \frac{p}{p-1}} \biggr) \biggr)^{\frac{p-1}{p}}. \end{aligned}$$

This ends the proof. □

### Remark 19

In Corollary 17, if we choose

- $\eta (b,a)=b-a$ and $\varphi =0$, we get
  $$\begin{aligned} & \biggl\vert \frac{f(a)+f(b)}{2}-\frac{\varGamma (\alpha +1))}{2(b-a)^{\alpha }} \bigl[J_{a^{+}}^{\alpha }f(b)+J_{b^{-}}^{\alpha }f(a) \bigr] \biggr\vert \\ &\quad \leq \biggl(\frac{(b-a)^{2}}{2(\alpha +1)} \biggr) \biggl(\frac{2^{ \alpha }-1}{2^{\alpha }} \biggr)^{\frac{1}{p}} \biggl( \biggl[\frac{ \pi }{2}-\beta \biggl( \frac{1}{2},\alpha +\frac{5}{2} \biggr) -\beta \biggl( \frac{3}{2},\alpha +\frac{3}{2} \biggr) \biggr]\\ &\qquad {}\times \biggl( \bigl\vert f''(a) \bigr\vert ^{\frac{p}{p-1}}+\frac{1-\lambda }{\lambda } \bigl\vert f''(b) \bigr\vert ^{\frac{p}{p-1}} \biggr) \biggr)^{\frac{p-1}{p}}. \end{aligned}$$
- $\eta (b,a)=b-a$, $\varphi =0$, and $\lambda =\frac{1}{2}$, we get
  $$\begin{aligned} & \biggl\vert \frac{f(a)+f(b)}{2}-\frac{\varGamma (\alpha +1))}{2(b-a)^{\alpha }} \bigl[J_{a^{+}}^{\alpha }f(b)+J_{b^{-}}^{\alpha }f(a) \bigr] \biggr\vert \\ &\quad \leq \biggl(\frac{(b-a)^{2}}{2(\alpha +1)} \biggr) \biggl(\frac{2^{ \alpha }-1}{2^{\alpha }} \biggr)^{\frac{1}{p}} \biggl( \biggl[\frac{ \pi }{2}-\beta \biggl( \frac{1}{2},\alpha +\frac{5}{2} \biggr) -\beta \biggl( \frac{3}{2},\alpha +\frac{3}{2} \biggr) \biggr]\\ &\qquad {}\times \bigl( \bigl\vert f''(a) \bigr\vert ^{\frac{p}{p-1}}+ \bigl\vert f''(b) \bigr\vert ^{\frac{p}{p-1}} \bigr) \biggr) ^{\frac{p-1}{p}}. \end{aligned}$$

### Corollary 18

*Under the assumptions of Theorem *14. *If* $|f''|^{ \frac{p}{p-1}}$ *is* *h*-*convex*, *we have*

$$\begin{aligned} & \biggl\vert \frac{f(a)+f (a+e^{i \varphi } \eta (b,a) )}{2}-\frac{ \varGamma (\alpha +1))}{2 (e^{i \varphi }\eta (b,a) )^{\alpha }} \bigl[J_{a^{+}}^{\alpha }f \bigl(a+e^{i \varphi }\eta (b,a) \bigr) +J_{ (a+e^{i \varphi }\eta (b,a) )^{-}}^{\alpha }f(a) \bigr] \biggr\vert \\ &\quad \leq \biggl(\frac{ (e^{i \varphi }\eta (b,a) )^{2}}{2( \alpha +1)} \biggr)\biggl(\frac{2^{\alpha }-1}{2^{\alpha }} \biggr) ^{\frac{1}{p}} \\ &\qquad {}\times \biggl( \int _{0}^{1} h(t) \bigl\vert (1-t)^{\alpha }-t^{ \alpha } \bigr\vert \,dt \biggr)^{\frac{p-1}{p}}\biggl( \bigl\vert f'(a) \bigr\vert ^{\frac{p}{p-1}}+\frac{1-\lambda }{\lambda } \bigl\vert f'(b) \bigr\vert ^{ \frac{p}{p-1}} \biggr)^{\frac{p-1}{p}}. \end{aligned}$$

### Proof

Using (9), by Theorem 14, it can be proved easily. It is omitted. □

## Conclusion

In the present paper, using the notion of *F* and *F*-convex function (see [17]), we construct some new inequalities of Hermite–Hadamard type for differentiable function via Riemann–Liouville fractional integral. We also established some trapezoid type inequalities for a function of whose second derivatives absolutely values are *F*-convex. Moreover, we obtained some new inequalities of Hermite–Hadamard type for Riemann–Liouville fractional integrals and via classical integrals. The results presented in this paper would provide generalizations and extension of those given in earlier work.
